# Prevalence of active convulsive epilepsy in sub-Saharan Africa and associated risk factors: cross-sectional and case-control studies

**DOI:** 10.1016/S1474-4422(13)70003-6

**Published:** 2013-03-13

**Authors:** Anthony K Ngugi, Christian Bottomley, Immo Kleinschmidt, Ryan G Wagner, Angelina Kakooza-Mwesige, Kenneth Ae-Ngibise, Seth Owusu-Agyei, Honorati Masanja, Gathoni Kamuyu, Rachael Odhiambo, Eddie Chengo, Josemir W Sander, Charles R Newton, for the SEEDS group

**Affiliations:** a Studies of Epidemiology of Epilepsy in Demographic Surveillance Systems, International Network for the Demographic Evaluation of Populations and Their Health (INDEPTH), Accra, Ghana; b Kenya Medical Research Institute/Wellcome Trust Research Programme, Centre for Geographic Medicine Research–Coast, Kilifi, Kenya; c Department of Infectious Disease Epidemiology, London School of Hygiene and Tropical Medicine, London, UK; d MRC Tropical Epidemiology Group, London School of Hygiene and Tropical Medicine, London, UK; e Faculty of Epidemiology and Population Health, and Clinical Research Unit, London School of Hygiene and Tropical Medicine, London, UK; f MRC/Wits Rural Public Health and Health Transitions Research Unit (Agincourt), School of Public Health, Faculty of Health Sciences, University of the Witwatersrand, Johannesburg, South Africa; g Epidemiology and Public Health, Department of Public Health and Clinical Medicine and Department of Pharmacology and Clinical Neuroscience, Umeå University, Umeå, Sweden; h Iganga-Mayuge Health and Demographic Surveillance System, Iganga, Uganda; i Department of Paediatrics and Child Health, Makerere University College of Health Sciences, Kampala, Uganda; j Kintampo Health Research Centre, Kintampo, Ghana; k Ifakara Health Institute, Ifakara, Tanzania; l Department of Clinical and Experimental Epilepsy, UCL Institute of Neurology, University College London, London, UK; m Neurosciences Unit, UCL Institute of Child Health, University College London, London, UK; n Stichting Epilepsie Instellingen Nederland, Heemstede, Netherlands; o Department of Psychiatry, University of Oxford, Oxford, UK

## Abstract

**Background:**

The prevalence of epilepsy in sub-Saharan Africa seems to be higher than in other parts of the world, but estimates vary substantially for unknown reasons. We assessed the prevalence and risk factors of active convulsive epilepsy across five centres in this region.

**Methods:**

We did large population-based cross-sectional and case-control studies in five Health and Demographic Surveillance System centres: Kilifi, Kenya (Dec 3, 2007–July 31, 2008); Agincourt, South Africa (Aug 4, 2008–Feb 27, 2009); Iganga-Mayuge, Uganda (Feb 2, 2009–Oct 30, 2009); Ifakara, Tanzania (May 4, 2009–Dec 31, 2009); and Kintampo, Ghana (Aug 2, 2010–April 29, 2011). We used a three-stage screening process to identify people with active convulsive epilepsy. Prevalence was estimated as the ratio of confirmed cases to the population screened and was adjusted for sensitivity and attrition between stages. For each case, an age-matched control individual was randomly selected from the relevant centre's census database. Fieldworkers masked to the status of the person they were interviewing administered questionnaires to individuals with active convulsive epilepsy and control individuals to assess sociodemographic variables and historical risk factors (perinatal events, head injuries, and diet). Blood samples were taken from a randomly selected subgroup of 300 participants with epilepsy and 300 control individuals from each centre and were screened for antibodies to *Toxocara canis, Toxoplasma gondii, Onchocerca volvulus, Plasmodium falciparum, Taenia solium*, and HIV. We estimated odds ratios (ORs) with logistic regression, adjusted for age, sex, education, employment, and marital status.

**Results:**

586 607 residents in the study areas were screened in stage one, of whom 1711 were diagnosed as having active convulsive epilepsy. Prevalence adjusted for attrition and sensitivity varied between sites: 7·8 per 1000 people (95% CI 7·5–8·2) in Kilifi, 7·0 (6·2–7·4) in Agincourt, 10·3 (9·5–11·1) in Iganga-Mayuge, 14·8 (13·8–15·4) in Ifakara, and 10·1 (9·5–10·7) in Kintampo. The 1711 individuals with the disorder and 2032 control individuals were given questionnaires. In children (aged <18 years), the greatest relative increases in prevalence were associated with difficulties feeding, crying, or breathing after birth (OR 10·23, 95% CI 5·85–17·88; p<0·0001); abnormal antenatal periods (2·15, 1·53–3·02; p<0·0001); and head injury (1·97, 1·28–3·03; p=0·002). In adults (aged ≥18 years), the disorder was significantly associated with admission to hospital with malaria or fever (2·28, 1·06–4·92; p=0·036), exposure to *T canis* (1·74, 1·27–2·40; p=0·0006), exposure to *T gondii* (1·39, 1·05–1·84; p=0·021), and exposure to *O volvulus* (2·23, 1·56–3·19; p<0·0001). Hypertension (2·13, 1·08–4·20; p=0·029) and exposure to *T solium* (7·03, 2·06–24·00; p=0·002) were risk factors for adult-onset disease.

**Interpretation:**

The prevalence of active convulsive epilepsy varies in sub-Saharan Africa and that the variation is probably a result of differences in risk factors. Programmes to control parasitic diseases and interventions to improve antenatal and perinatal care could substantially reduce the prevalence of epilepsy in this region.

**Funding:**

Wellcome Trust, University of the Witwatersrand, and South African Medical Research Council.

## Introduction

Epilepsy is one of the most common neurological conditions worldwide. The prevalence of epilepsy is highest in poor countries^1^ and in rural areas,^2^ particularly in sub-Saharan Africa.^3^ Reported prevalence varies between studies in sub-Saharan Africa,^3^ but the cause of this variation is unknown. Differences in methodology and case definition could partly explain this heterogeneity, but the epidemiology of parasitic diseases (particularly malaria,^4^ cysticercosis,^5^, ^6^, ^7^, ^8^ onchocerciasis,^9^, ^10^ toxocariasis,^6^, ^11^ and toxoplasmosis^12^), perinatal events,^13^ head injuries,^14^ HIV infection,^15^ and hereditary factors^16^ might also contribute. Previous studies in sub-Saharan Africa have focused on a small number of risk factors in areas with high prevalence of epilepsy,^3^ but none have examined a wide range of potential risk factors. We established the prevalence of active convulsive epilepsy and its risk factors in sub-Saharan Africa, and assessed the relative contributions of parasitic and non-parasitic risk factors for epilepsy in this region.

## Methods

### Study design

We did large population-based cross-sectional and case-control studies in five Health and Demographic Surveillance System (HDSS) centres that are part of the International Network for the Demographic Evaluation of Populations and Their Health (INDEPTH). Surveys were done in Kilifi, Kenya (Dec 3, 2007–July 31, 2008); Agincourt, South Africa (Aug 4, 2008–Feb 27, 2009); Iganga-Mayuge, Uganda (Feb 2, 2009–Oct 30, 2009); Ifakara, Tanzania (May 4, 2009–Dec 31, 2009); and Kintampo, Ghana (Aug 2, 2010–April 29, 2011). We selected these centres because of the endemicity of parasitic diseases associated with epilepsy, availability of health facilities able to provide support to people with epilepsy, and logistics.

All aspects of the study were approved by the ethics committees of University College London and the London School of Hygiene and Tropical Medicine, and by the ethics review boards in each of the participating countries. All participants or guardians gave written informed consent.

### Participants and procedures

We used a three-stage screening process to identify cases of active convulsive epilepsy.^14^ In the first stage, two screening questions were asked during a routine, door-to-door census organised by each HDSS centre. Heads of households were interviewed about whether any residents had had convulsions. In the second stage, trained lay fieldworkers administered a detailed questionnaire^17^ (appendix) to individuals identified as having a history of convulsions in stage one. Individuals whose responses to the questionnaire suggested they might have epilepsy were examined during stage three by clinicians who made a final diagnosis.

To enable comparison between our three-stage method and the two-stage surveys used in other population-based studies in Africa,^18^, ^19^, ^20^, ^21^, ^22^ we selected a random population sample from each centre's census database with the RAND() command in MySQL (Oracle, Redwood Shores, CA, USA). The questionnaire^17^ used in the second stage of the study was administered to this randomly sampled population; individuals identified as possibly having epilepsy after the questionnaire results were assessed clinically in stage three.

We studied only convulsive epilepsies, because convulsions are the most reliably detected and reported symptom, and are associated with the greatest morbidity (eg, burns and stigma) and mortality.^23^ Active convulsive epilepsy was defined as two or more unprovoked convulsive seizures (which could be primarily or secondarily generalised) occurring at least 24 h apart, with at least one seizure in the preceding 12 months.^14^, ^24^

For each case, an age-matched control individual was randomly selected from the relevant centre's census database with the RAND() command. The control individuals were frequency matched by age groups: 0–5 years, 6–12 years, 13–18 years, 19–28 years, 29–49 years, and 50 years or older. In the case-control study, two or three control individuals were selected to compensate for non-response and ensure balance in the number of cases and control individuals at each centre. All control individuals were assessed by a clinician to confirm that they did not have epilepsy.

Fieldworkers then administered questionnaires based on those used in previous studies^25^, ^26^, ^27^ to individuals identified as having epilepsy and control individuals. Fieldworkers, who were masked to the status (case or control) of the person they were interviewing, gathered data on sociodemographic variables and historical risk factors (perinatal events, head injuries, and diet). Clinical history was also obtained by masked, trained clinicians (the same clinicians who made initial diagnoses) and they made a diagnosis of active convulsive epilepsy.

When the study participants were younger than 18 years or had cognitive impairment, the mother or caregiver was interviewed. The questionnaires administered to mothers or caregivers included questions about antenatal (eg, severe abdominal pain, vaginal bleeding, or infection during pregnancy) and perinatal events (difficulties breathing, feeding, or crying after birth, as recalled by the mother or caregiver^28^). Questions about consumption of alcohol and use of recreational drugs were administered to adult participants only.

Blood samples were taken from a subgroup of 300 participants with epilepsy and 300 control individuals from each centre who were randomly selected with the RAND() command. This sample size would allow detection of an odds ratio (OR) greater than 2·4, with 80% power and the assumption that 5% of control individuals had epilepsy. The samples were screened for antibodies to *Toxocara canis, Toxoplasma gondii, Onchocerca volvulus, Plasmodium falciparum, Taenia solium*, and HIV. Exposure was established by detection of IgG antibodies to the parasitic antigens. IgG antibodies against *T canis* were detected with a commercial kit (*Toxocara* IgG-ELISA, Cypress Diagnostics, Belgium; sensitivity 97%; specificity 78%). Anti-*Toxocara* IgG4 antibodies with an optical density greater than the cutoff (mean plus three standard deviations of 30 IgG-negative serum samples) were interpreted as positive. IgG antibodies against *T gondii* were detected with a commercial kit (*Toxoplasma* IgG-ELISA, Genesis Diagnostics, Ely, UK; 100% agreement with test samples) and were judged positive when optical density was greater than that of the positive 8 IU/mL sample in the kit. Exposure to *O volvulus* was established with a modification of an ELISA that detects IgG4 to the recombinant antigen Ov-16GST (sensitivity 90%; specificity 98%).^29^ A sample with an optical density greater than the cutoff (mean plus three standard deviations of 30 serum samples from the Agincourt HDSS, where onchocerciasis is not prevalent) were interpreted as positive. Exposure to malaria was established with an in-house ELISA^30^ that tests for IgG antibodies to crude schizont extract from a *P falciparum* A4 clone line, which is derived from a laboratory strain. Exposure to the larval stage (cysticercosis) and adult stage (taeniasis) of the parasite *T solium* was established with a western blot (sensitivity 97%; specificity 99%; detection of cases with two or more viable cysts in the brain) and antibodies to taeniasis (RES33 antigen; sensitivity 99%; specificity 93%).^31^ IgG antibodies to HIV type 1 or type 2, or both, were detected with the fourth-generation screening test Vironostika HIV Uniform II Ag/Ab (BioMerieux, France) according to the manufacturer's instructions.

### Statistical analysis

We double entered and verified all data in the MySQL open-source database (version 5; Oracle Corporation, Redwood Shores, CA, USA). All statistical analyses were done in STATA (version 12).

We estimated an unadjusted prevalence of the disorder with 95% CIs for each centre by dividing the number of cases confirmed in stage three by the total number of individuals screened in stage one. We used multiple imputation^32^, ^33^ to reduce bias because of attrition between stages of the survey. Imputation (using the ice command in Stata) was done separately for each centre and five imputed datasets were created.^32^, ^34^ We further adjusted prevalence estimates by dividing by the estimated sensitivity (0·486 for the three-stage method; 0·767 for the two-stage method).^35^ To account for differences in age distribution between study centres, we produced age-standardised, centre-specific prevalence estimates, with the age distribution of the US population in 2000 as reference.^36^ We used the US population to enable comparison between our study and others in countries of low and middle income, in which the US population has been used for adjustment. We used a logistic regression model to test for heterogeneity between study centres after adjusting for age and sex.

For the case-control study, adjusted ORs were obtained by fitting logistic regression models that included age, sex, education (none, primary, or secondary and above), employment, marital status, and country as covariates as well as the risk factor of interest. Age was modelled as a fractional polynomial of degree two.^37^ In models fitted to data for children, the marital status of the mother and employment status of the mother and father were included as covariates. For variables for which an event date was available (head injuries in adults and hospital admissions for malaria or fever in adults and children), events after the seizure date or after an index date in control individuals were excluded. We selected index dates for each control individual by randomly sampling the dates of first seizure in individuals with epilepsy from the same age group with the bsample command in STATA.

Nutritional status was established with age-specific height and weight.^38^ Adults (older than 19 years) with a body-mass index less than 18·5 kg/m^2^, adolescents (aged 10–19 years) with body-mass-index-for-age *Z* scores of less than −2, and children (younger than 10 years) with weight-for-height or height-for-age *Z* scores of less than −2 were deemed to be malnourished. All *Z* scores were computed with the zanthro command in Stata. The 2000 US growth charts were used as the reference distribution.^39^

Population attributable fractions (PAFs) were estimated separately for every risk factor and for combinations of risk factors with the user-written command punacc, which implements the method of Greenland and Drescher.^40^ No adjustment for multiple comparisons was made, because this adjustment can lead to errors of interpretation when strong previous evidence of association is available for several risk factors.^41^

### Role of the funding source

The sponsors had no role in study design, data collection, data analysis, data interpretation, or writing of the report. All authors had full access to all the data in the study and the corresponding author had final responsibility for the decision to submit for publication.

## Results

586 607 residents in the study areas were screened in stage one, of whom 1711 were diagnosed as having active convulsive epilepsy by the end of stage three (table 1). The proportion of individuals identified as having convulsive seizures in stage one was highest in Iganga-Mayuge, Uganda, and lowest in Agincourt, South Africa (table 1). The final crude prevalence was highest in Ifakara, Tanzania, and was lowest in Kintampo, Ghana (table 1). After adjustment for attrition between stages one and two, and between stages two and three, by multiple imputation, we noted that prevalence doubled in Iganga-Mayuge and in Kintampo, and increased by more than 1·5 times in Ifakara (table 1). After adjustment for both attrition and sensitivity of the three-stage method, prevalence was lowest in Agincourt and highest in Ifakara, with a significant difference between the two (table 1). The age-standardised prevalence was lowest in Iganga-Mayuge and highest in Ifakara (table 1).

**Table 1:** Prevalence of active convulsive epilepsy in five centres in sub-Saharan Africa

	**Population**	**Screened in stage one**	**Identified during screening in stage one**	Screened in stage two*	**Identified in stage two**	Screened in stage three*	**Diagnosed in stage three**	**Crude prevalence per 1000 people**	**Prevalence per 1000 people adjusted for attrition**	**Prevalence per 1000 people adjusted for attrition and sensitivity**	**Prevalence ratios**†	**Age-standardised prevalence per 1000 people**‡
Kilifi, Kenya	233 881	232 176 (99·3%)	5152 (2·2%)	4886 (94·8%)	1123 (23·0%)	948 (84·4%)	699 (73·7%)	3·0 (2·8–3·2)	3·8 (3·5–4·0)	7·8 (7·5–8·2)	1·0 (NA)	7·4 (7·1–7·8)
Agincourt, South Africa	83 121	82 818 (99·6%)	546 (0·7%)	515 (94·3%)	354 (68·7%)	328 (92·7%)	245 (74·7%)	3·0 (2·6–3·3)	3·4 (3·0–3·8)	7·0 (6·2–7·4)	0·9 (0·8–1·1)	8·1 (7·5–8·7)
Iganga-Mayuge, Uganda	69 186	64 172 (92·8%)	4917 (7·7%)	3145 (64·0%)	500 (15·9%)	321 (64·2%)	152 (47·4%)	2·4 (2·0–2·8)	5·0 (4·4–5·6)	10·3 (9·5–11·1)	1·8 (1·7–1·9)	6·8 (6·2–7·5)
Ifakara, Tanzania	104 889	93 645 (89·3%)	1389 (1·5%)	1321 (95·1%)	528 (40·0%)	481 (91·1%)	366 (76·1%)	3·9 (3·5–4·3)	7·2 (6·5–7·8)	14·8 (13·8–15·4)	1·4 (1·3–1·5)	15·5 (14·7–16·3)
Kintampo, Ghana	129 812	113 796 (87·7%)	3344 (2·9%)	3046 (91·1%)	570 (18·7%)	443 (77·7%)	249 (56·2%)	2·2 (1·9–2·5)	4·9 (4·4–5·3)	10·1 (9·5–10·7)	0·8 (0·8–0·9)	10·1 (9·5–10·7)
Overall	620 889	586 607 (94·5%)	15 348 (2·6%)	12 913 (84·1%)	3075 (23·8%)	2521 (82·0%)	1711 (67·9%)	..	..	..	..	..

Data in parentheses are % or 95% CI. NA=not applicable.

* Expressed as a percentage of the total identified during screening in the preceding stage.

† On the basis of estimates adjusted for attrition and sensitivity; p=0·006.

‡ Standardised to the age distribution of the US population in 2000 (based on estimates adjusted for attrition and sensitivity of the three-stage method).

Age-specific prevalence varied between centres (figure). In all centres, most cases of epilepsy occurred in children (figure). Prevalence in Kilifi, Kenya, was 30% higher in male residents than in female ones and 60% higher in adolescents and young adults aged 13–28 years than in the youngest age group (0–5 years; table 2). In Kintampo, prevalence was marginally higher in male residents than in female ones, and varied significantly with age (table 2). In the other centres, prevalence varied by age but not by sex (table 2).

**Figure:**
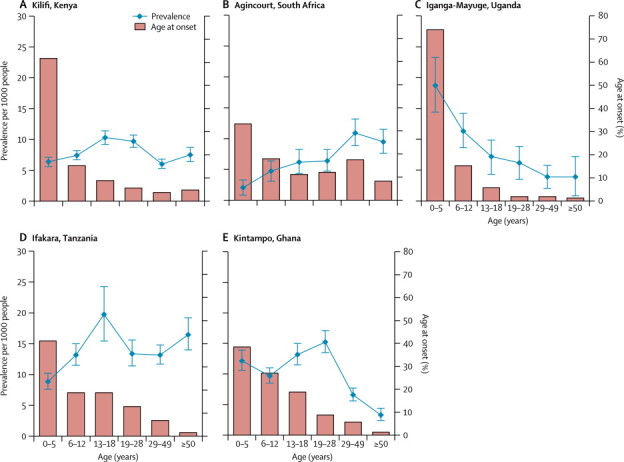
Age-specific prevalence and age at onset of active convulsive epilepsy in the five centres

**Table 2:** Age-specific and sex-specific prevalence of active convulsive epilepsy by centre

		**Participants screened**	Prevalence per 1000 people (95% CI)*	**Univariable analysis**		**Multivariable analysis**	
				Prevalence ratio (95% CI)	p value	Prevalence ratio (95% CI)	p value
**Kilifi, Kenya**							
Sex							
	Female	123 914	7·0 (6·6–7·5)	1·0 (NA)	<0·0001	1·0 (NA)	0·0004
	Male	108 262	8·6 (8·1–9·2)	1·3 (1·1–1·5)	..	1·3 (1·1–1·4)	..
Age (years)							
	0–5	41 868	6·3 (5·6–7·1)	1·0 (NA)	<0·0001	1·0 (NA)	0·0007
	6–12	52 248	7·4 (6·7–8·2)	1·2 (1·3–1·8)	..	1·2 (0·9–1·5)	..
	13–18	34 228	10·3 (9·2–11·4)	1·6 (1·5–1·8)	..	1·6 (1·3–2·1)	..
	19–28	37 523	9·7 (8·7–10·7)	1·6 (1·4–1·4)	..	1·6 (1·3–2·0)	..
	29–49	42 269	6·0 (5·3–6·8)	1·0 (0·9–1·1)	..	1·0 (0·8–1·3)	..
	≥50	24 040	7·5 (6·4–8·7)	1·1 (1·0–1·2)	..	1·1 (0·8–1·5)	..
**Agincourt, South Africa**							
Sex							
	Female	43 065	6·6 (5·3–7·6)	1·0 (NA)	0·369	1·0 (NA)	0·086
	Male	39 753	7·6 (6·2–8·6)	1·1 (0·9–1·4)	..	1·2 (1·0–1·6)	..
Age (years)							
	0–5	11 531	2·1 (0·8–3·3)	1·0 (NA)	<0·0001	1·0 (NA)	0·0003
	6–12	13 044	4·7 (3·1–6·4)	2·4 (1·2–4·8)	..	2·6 (1·3–5·3)	..
	13–18	12 341	6·2 (4·3–8·2)	3·2 (1·7–6·3)	..	3·2 (1·6–6·4)	..
	19–28	17 770	6·4 (4·7–8·2)	3·3 (1·7–6·3)	..	3·1 (1·5–6·0)	..
	29–49	18 491	10·9 (8·8–13·2)	5·5 (3·0–10·3)	..	5·5 (2·8–10·5)	..
	≥50	9641	9·5 (7·6–11·5)	4·9 (2·5–9·5)	..	4·7 (2·3–9·4)	..
**Iganga-Mayuge, Uganda**							
Sex							
	Female	32 518	8·9 (8·0–10·0)	1·0 (NA)	0·871	1·0 (NA)	0·189
	Male	31 625	9·6 (8·5–10·7)	1·0 (0·8–1·3)	..	1·3 (0·9–1·7)	..
Age (years)							
	0–5	11 444	18·7 (14·4–23·3)	1·0 (NA)	<0·0001	1·0 (NA)	0·0001
	6–12	14 972	11·3 (8·6–14·2)	0·5 (0·4–0·7)	..	0·9 (0·9–1·4)	..
	13–18	10 722	7·2 (4·3–9·9)	0·3 (0·2–0·5)	..	0·8 (0·5–1·4)	..
	19–28	10 509	6·2 (3·5–8·8)	0·3 (0·2–0·5)	..	0·6 (0·4–1·1)	..
	29–49	11 765	3·9 (2·1–5·8)	0·2 (0·1–0·3)	..	0·3 (0·2–0·6)	..
	≥50	4740	3·9 (0·8–7·2)	0·2 (0·1–0·4)	..	0·2 (0·1–0·7)	..
**Ifakara, Tanzania**							
Sex							
	Female	44 694	14·0 (12·1–15·8)	1·0 (NA)	0·191	1·0 (NA)	0·467
	Male	44 245	10·9 (10·9–14·0)	0·9 (0·8–1·1)	..	0·9 (0·8–1·1)	..
Age (years)							
	0–5	21 028	8·8 (7·6–10·2)	1·0 (NA)	<0·0001	1·0 (NA)	0·0002
	6–12	17 655	13·2 (11·5–15·0)	1·4 (1·1–1·8)	..	2·1 (1·4–3·1)	..
	13–18	9383	19·8 (17·0–22·9)	2·2 (1·7–3·0)	..	3·8 (2·6–5·5)	..
	19–28	13 223	13·4 (11·4–15·6)	1·4 (1·0–1·9)	..	2·4 (1·6–3·5)	..
	29–49	21 866	13·2 (11·7–14·8)	1·4 (1·1–1·8)	..	2·0 (1·4–2·9)	..
	≥50	10 272	16·5 (14·0–19·2)	1·8 (1·4–2·5)	..	1·0 (0·6–1·7)	..
**Kintampo, Ghana**							
Sex							
	Female	57 880	9·1 (7·8–10·3)	1·0 (NA)	0·027	1·0 (NA)	0·057
	Male	55 904	11·1 (9·7–12·3)	1·2 (1·0–1·4)	..	1·2 (1·0–1·4)	..
Age (years)							
	0–5	17 424	12·1 (10·6–13·9)	1·0 (NA)	<0·0001	1·0 (NA)	0·0009
	6–12	23 121	9·7 (8·5–11·0)	0·8 (0·6–1·0)	..	0·8 (0·6–1·0)	..
	13–18	17 019	13·2 (11·5–15·0)	1·1 (0·9–1·5)	..	1·1 (0·9–1·5)	..
	19–28	18 843	15·2 (13·5–17·1)	1·2(1·0–1·6)	..	1·2 (1·0–1·6)	..
	29–49	23 091	6·6 (5·6–7·7)	0·5(0·4–0·7)	..	0·5 (0·4–0·7)	..
	≥50	14 286	3·3 (2·4–4·4)	0·2 (0·1–0·4)	..	0·2 (0·1–0·4)	..

NA=not applicable.

* On the basis of the number of cases estimated after adjustment for loss to follow-up and sensitivity of the screening method.

Prevalence estimates from the two-stage method were 20–49% higher than those from the three-stage method for Kilifi, Agincourt, Kintampo, and Iganga-Mayuge when the data were adjusted for attrition between stages and sensitivity of the screening methods (appendix). In Ifakara, adjusted prevalence was higher with the three-stage than the two-stage method (appendix).

The 1711 individuals identified as having active convulsive epilepsy in stage three and 2032 control individuals were given questionnaires to assess risk factors (Table 3, Table 4). 1659 control individuals refused to take part: 711 (57%) of 1238 in Kilifi, 259 (50%) of 520 in Agincourt, 298 (55%) of 537 in Iganga-Mayuge, and 391 (51%) of 772 in Kintampo (data from Ifakara are unavailable). Age matching resulted in a similar age distribution in case and control individuals (appendix). Individuals with active convulsive epilepsy were more likely to be male than were control individuals (51% *vs* 46%; p=0·003) and to have had no education (38% *vs* 32%; p<0·0001; appendix).

**Table 3:** Risk factors for active convulsive epilepsy in children (aged <18 years)

	Control individuals*	**Individuals with active convulsive epilepsy**†	**Odds ratio (95% CI)**‡	**p value**
Seizures in the family	122/1028 (11·9%)	153/825 (18·5%)	1·72 (1·31–2·25)	<0·0001
Maternal seizures	9/1031 (0·9%)	19/822 (2·3%)	2·84 (1·24–6·49)	0·013
Abnormal delivery	39/1006 (3·9%)	47/816 (5·8%)	1·56 (0·98–2·47)	0·061
Abnormal antenatal period	66/1004 (6·6%)	118/797 (14·8%)	2·15 (1·53–3·02)	<0·0001
Home delivery	510/1007 (50·6%)	497/819 (60·7%)	1·24 (0·97–1·57)	0·081
Difficulties feeding, crying, or breathing	16/1003 (1·6%)	96/794 (12·1%)	10·23 (5·85–17·88)	<0·0001
Any other problems after birth	35/1014 (3·5%)	86/816 (10·5%)	2·77 (1·82–4·23)	<0·0001
Head injury	46/1025 (4·5%)	66/822 (8·0%)	1·97 (1·28–3·03)	0·002
Malnourished	194/952 (20·4%)	163/752 (21·7%)	1·00 (0·78–1·29)	0·992
Eats cassava	751/1029 (73·0%)	625/825 (75·8%)	0·99 (0·77–1·26)	0·913
Dogs in household	512/1031 (49·7%)	340/826 (41·2%)	0·91 (0·73–1·15)	0·441
Cats in household	502/1029 (48·8%)	403/823 (49·0%)	1·26 (0·98–1·62)	0·066
Eats pork	387/1025 (37·8%)	246/814 (30·2%)	0·99 (0·79–1·25)	0·939
Positive for malaria IgG (schizont)	491/610 (80·5%)	333/423 (78·7%)	1·14 (0·75–1·73)	0·535
Admitted to hospital with malaria or fever, or both	24/1036 (2·3%)	44/831 (5·3%)	2·01 (1·17–3·45)	0·011
Positive for *Toxocara canis* IgG4	123/510 (24·1%)	106/358 (29·6%)	1·19 (0·85–1·66)	0·316
Positive for *Toxoplasma gondii* IgG	150/599 (25·0%)	115/416 (27·6%)	1·15 (0·85–1·58)	0·367
Positive for *Taenia solium*	10/414 (2·4%)	5/243 (2·1%)	1·09 (0·34–3·45)	0·884
Positive for *Onchocerca volvulus*	61/414 (14·7%)	55/244 (22·5%)	1·67 (1·09–2·57)	0·019
HIV positive	50/606 (8·3%)	39/419 (9·3%)	1·28 (0·80–2·03)	0·304

Data are n/N (%), unless otherwise stated. N varies because data for each factor were obtained from varying numbers of participants. Data from all centres combined.

* Total n=1036.

† Total n=831.

‡ Odds ratio adjusted for age, sex, mother's education, mother's marital status, employment of mother or father, and country.

**Table 4:** Risk factors for active convulsive epilepsy in adults (aged ≥18 years)

	Control individuals*	**Individuals with active convulsive epilepsy**†	**Odds ratio (95% CI)**‡	**p value**
Seizures in the family	106/987 (10·7%)	180/869 (20·7%)	2·30 (1·73–3·07)	<0·0001
Maternal seizures	3/992 (0·3%)	11/873 (1·3%)	3·02 (0·75–12·14)	0·120
Abnormal delivery	37/898 (4·1%)	33/852 (3·9%)	1·11 (0·65–1·89)	0·706
Home delivery	571/841 (67·9%)	593/832 (71·3%)	1·18 (0·92–1·53)	0·196
Problems after birth	13/891 (1·5%)	62/841 (7·4%)	6·41 (3·28–12·53)	<0·0001
Head injury	18/996 (1·8%)	35/880 (4·0%)	2·29 (1·22–4·30)	0·010
Malnourished	127/960 (13·2%)	138/834 (16·5%)	1·25 (0·93–1·67)	0·138
Drinks alcohol	200/957 (20·9%)	143/855 (16·7%)	0·88 (0·67–1·15)	0·351
Eats cassava	750/991 (75·7%)	712/871 (81·7%)	1·46 (1·12–1·91)	0·005
Dogs in household	469/993 (47·2%)	356/875 (40·7%)	0·89 (0·71–1·12)	0·316
Cats in household	457/992 (46·1%)	403/876 (46·0%)	1·03 (0·80–1·32)	0·832
Eats pork	403/986) (40·9%)	348/873 (39·9%)	1·10 (0·89–1·36)	0·387
Uses drugs	73/963 (7·6%)	63/853 (7·4%)	1·21 (0·81–1·79)	0·357
Hypertension	21/983 (2·1%)	27/870 (3·1%)	1·74 (0·91–3·33)	0·094
Stroke	5/982 (0·5%)	10/872 (1·1%)	1·94 (0·61–6·15)	0·258
Diabetes mellitus	3/985 (0·3%)	6/873 (0·7%)	2·48 (0·56–10·87)	0·229
Positive for malaria IgG (schizont)	596/703 (84·8%)	473/563 (84·0%)	1·17 (0·75–1·81)	0·496
Admitted to hospital with malaria or fever, or both	11/996 (1·1%)	26/880 (3·0%)	2·28 (1·06–4·92)	0·036
Positive for *Toxocara canis* IgG4	160/611 (26·2%)	194/504 (38·5%)	1·74 (1·27–2·40)	0·0006
Positive for *Toxoplasma gondii* IgG	307/692 (44·4%)	265/555 (47·7%)	1·39 (1·05–1·84)	0·021
Positive for *Taenia solium*	8/421 (1·9%)	10/290 (3·4%)	1·98 (0·72–5·43)	0·183
Positive for *Onchocerca volvulus*	128/422 (30·3%)	147/291 (50·5%)	2·23 (1·56–3·19)	<0·0001
HIV positive	132/698 (18·9%)	101/558 (18·1%)	0·85 (0·61–1·18)	0·334

Data are n/N (%), unless otherwise stated. Data from all centres combined.

* Total n=996.

† Total n=880.

‡ Odds ratio adjusted for age, sex, education, marital status, employment, and country.

For children (aged <18 years), factors related to their antenatal period and delivery were associated with the greatest relative increase in prevalence of epilepsy (table 3). Seizures in the family, maternal seizures, and head injuries were also strong risk factors (table 3). Of parasitic risk factors, exposure to *O volvulus* and hospital admission for malaria or fever, or both, were significantly associated with the disorder (table 3).

Overall, parasitic risk factors were more strongly associated with active convulsive epilepsy in adults than in children (Table 3, Table 4). In adults, we recorded significant associations between the disorder and exposure to *T canis* and *T gondii* (table 4). The association between exposure to *T solium* and the disorder in adults was not significant (table 4), but we did record a strong association with adult-onset disease (table 5). Active convulsive epilepsy and HIV were not associated (Table 3, Table 4). Cassava consumption was associated with active convulsive epilepsy in adults but not in children (Table 3, Table 4). Hypertension was a significant risk factor for late-onset disease (table 5).

**Table 5:** Risk factors for adult-onset active convulsive epilepsy

	Control individuals*	**Individuals with adult-onset active convulsive epilepsy**†	**Odds ratio (95% CI)**‡	**p value**
Seizures in the family	106/987 (10·7%)	62/299 (20·7%)	2·45 (1·66–3·62)	<0·0001
Maternal seizures	3/992 (0·3%)	4/300 (1·3%)	4·69 (0·68–32·33)	0·117
Abnormal delivery	37/898 (4·1%)	8/287 (2·8%)	0·84 (0·36–1·96)	0·685
Home delivery	571/841 (67·9%)	210/278 (75·5%)	0·99 (0·68–1·46)	0·976
Problems after birth	13/891 (1·5%)	10/281 (3·6%)	3·46 (1·36–8·78)	0·009
Head injury	35/996 (3·5%)	23/304 (7·6%)	2·00 (1·07–3·76)	0·030
Malnourished	127/960 (13·2%)	52/291 (17·9%)	1·21 (0·82–1·80)	0·343
Drinks alcohol	200/957 (20·9%)	79/299 (26·4%)	0·99 (0·70–1·40)	0·958
Eats cassava	750/991 (75·7%)	231/302 (76·5%)	1·21 (0·85–1·73)	0·287
Dogs in household	469/993 (47·2%)	123/301 (40·9%)	0·99 (0·71–1·37)	0·936
Cats in household	457/992 (46·1%)	142/303 (46·9%)	1·21 (0·84–1·75)	0·297
Eats pork	403/986 (40·9%)	129/302 (42·7%)	1·07 (0·79–1·44)	0·679
Uses drugs	73/963 (7·6%)	32/295 (10·8%)	1·11 (0·67–1·82)	0·690
Hypertension	21/983 (2·1%)	21/301 (7·0%)	2·13 (1·08–4·20)	0·029
Stroke	5/982 (0·5%)	6/300 (2·0%)	1·90 (0·54–6·73)	0·321
Diabetes mellitus	3/985 (0·3%)	5/301 (1·7%)	2·70 (0·60–12·08)	0·194
Positive for malaria IgG (schizont)	596/703 (84·8%)	168/201 (83·6%)	1·29 (0·71–2·36)	0·402
Admitted to hospital with malaria or fever, or both	17/996 (1·7%)	11/304 (3·6%)	3·27 (1·37–7·82)	0·008
Positive for *Toxocara canis* IgG4	160/611 (26·2%)	58/176 (33·0%)	1·76 (1·09–2·84)	0·02
Positive for *Toxoplasma gondii* IgG	307/692 (44·4%)	97/195 (49·7%)	1·34 (0·89–2·01)	0·158
Positive for *Taenia solium*	8/421 (1·9%)	9/99 (9·1%)	7·03 (2·06–24·00)	0·002
Positive for *Onchocerca volvulus*	128/422 (30·3%)	47/98 (48·0%)	2·10 (1·24–3·57)	0·006
HIV positive	132/698 (18·9%)	42/199 (21·1%)	0·93 (0·59–1·46)	0·751

Data are n/N (%), unless otherwise stated. Data from all centres combined. For adult-onset cases, the first seizure occurs later, so we used an index date for control individuals that was different from that in other analyses.

* Total n=996.

† Onset when older than 18 years; total n=304.

‡ Odds ratio adjusted for age, sex, education, marital status, employment, and country.

Associations were generally consistent across study sites (appendix). However, the interaction with study site in children was significant for adverse perinatal events (p=0·0068) and a history of seizures in the family (p=0·0112). In adults, the interaction was significant for a history of seizures in the family (p=0·0096), cassava consumption (p=0·0008), pork consumption (p=0·0099), and alcohol consumption (p=0·0352).

With the exception of women who delivered their babies at home and participants who ate cassava or were malnourished, the proportion of participants exposed to non-parasitic risk factors was generally low (<10%) across all five centres (appendix). Most of the population consumed cassava and home delivery was also very common, particularly in Kilifi (83% of control individuals; 81% of case individuals) and Kintampo (86% of control individuals; 85% of case individuals; appendix). Malnutrition was prevalent at all centres (10–36% of participants; appendix). Head injuries were common among children in Kintampo (19% of control individuals; 23% of case individuals), but less so elsewhere (appendix).

Prevalence of parasitic risk factors was higher than was that of non-parasitic risk factors, especially in adults (appendix). However, we recorded substantial variation between centres: in adults, the proportion of control individuals exposed to *O volvulus* ranged from 9% in Iganga-Mayuge to 38% in Ifakara, to *T gondii* from 11% in Agincourt to 67% in Kintampo, and to *T canis* from 8% in Agincourt to 62% in Ifakara (appendix).

The associations between parasitic risk factors and focal active convulsive epilepsy in adults were of similar magnitude to those recorded for active convulsive epilepsy (appendix).

The PAFs for non-parasitic risk factors were generally low (Table 6, Table 7), although the total PAF associated with risk factors related to the antenatal and perinatal periods in children was 33% across all centres (table 6). PAFs for the parasitic risk factors were higher in adults than children (except hospital admission for malaria) and varied substantially between the centres (Table 6, Table 7). The combined PAF for parasitic risk factors in adults varied from 9% in Agincourt to 62% in Ifakara (table 7).

**Table 6:** Population attributable fractions associated with each risk factor in children (aged <18 years)

	**All centres**	**Kilifi, Kenya**	**Agincourt, South Africa**	**Iganga-Mayuge, Uganda**	**Ifakara, Tanzania**	**Kintampo, Ghana**
Seizures in the family	0·08 (0·04 to 0·12)	0·07 (0·03–0·10)	0·06 (0·01 to 0·10)	0·09 (0·04–0·15)	0·06 (0·02 to 0·10)	0·14 (0·06 to 0·22)
Maternal seizures	0·01 (0 to 0·03)	0·01 (0 to 0·02)	0·02 (−0·01 to 0·05)	0·02 (0 to 0·05)	0·02 (0 to 0·04)	0·02 (−0·01 to 0·04)
Abnormal delivery	0·02 (0 to 0·04)	0·02 (0 to 0·05)	0·04 (−0·01 to 0·08)	0·01 (−0·01 to 0·03)	0·01 (0 to 0·02)	0·03 (−0·01 to 0·08)
Abnormal antenatal period	0·08 (0·04 to 0·11)	0·11 (0·06 to 0·16)	0·04 (0 to 0·08)	0·05 (0·02 to 0·09)	0·04 (0·01 to 0·06)	0·08 (0·02 to 0·13)
Home delivery	0·12 (−0·01 to 0·23)	0·16 (−0·02 to 0·32)	0·04 (−0·01 to 0·09)	0·05 (−0·01 to 0·10)	0·07 (−0·01 to 0·15)	0·15 (−0·02 to 0·29)
Difficulties feeding, crying, or breathing	0·11 (0·08 to 0·13)	0·07 (0·04 to 0·10)	0·11 (0·02 to 0·18)	0·06 (0·02 to 0·10)	0·10 (0·06 to 0·15)	0·34 (0·23 to 0·44)
Any other problems after birth	0·07 (0·04 to 0·09)	0·08 (0·05 to 0·12)	0·04 (0 to 0·08)	0·06 (0·02 to 0·10)	0·06 (0·02 to 0·09)	0·03 (0 to 0·06)
Head injury	0·04 (0·01 to 0·06)	0·04 (0·01 to 0·06)	0·02 (−0·01 to 0·04)	0·02 (0 to 0·05)	0·02 (0 to 0·03)	0·12 (0·03 to 0·19)
Malnourished	0 (−0·06 to 0·05)	0·00 (−0·07 to 0·06)	0 (−0·03 to 0·03)	0 (−0·06 to 0·06)	0 (−0·05 to 0·05)	0 (−0·03 to 0·03)
Cats in household	0·10 (0 to 0·20)	0·11 (−0·01 to 0·22)	0 (NA)	0 (0 to 0·01)	0·20 (−0·01 to 0·36)	0·07 (−0·01 to 0·15)
Positive for malaria IgG (schizont)	0·10 (−0·24 to 0·34)	0·07 (−0·17 to 0·26)	0·03 (−0·07 to 0·12)	0·12 (−0·32 to 0·42)	0·12 (−0·31 to 0·41)	0·12 (−0·32 to 0·42)
Admitted to hospital with malaria or fever, or both	0·03 (0·01 to 0·05)	0·04 (0·01 to 0·08)	0 (0 to 0)	0 (NA)	0·03 (0 to 0·06)	0 (NA)
Positive for *Toxocara canis* IgG4	0·05 (−0·05 to 0·13)	0·06 (−0·06 to 0·16)	0·02 (−0·02 to 0·05)	0·03 (−0·03 to 0·08)	0·09 (−0·09 to 0·24)	0·02 (−0·03 to 0·07)
Positive for *Toxoplasma gondii* IgG	0·04 (−0·04 to 0·11)	0·03 (−0·03 to 0·08)	0 (NA)	0·03 (−0·03 to 0·08)	0·06 (−0·07 to 0·17)	0·06 (−0·07 to 0·17)
Positive for *Taenia solium*	0 (−0·01 to 0·01)	NA	NA	0 (−0·02 to 0·02)	0 (−0·02 to 0·02)	0 (−0·04 to 0·04)
Positive for *Onchocerca volvulus*	0·06 (0·01 to 0·10)	NA	NA	0·05 (0 to 0·10)	0·09 (0·01 to 0·17)	0·14 (0·01 to 0·25)
HIV positive	0·02 (−0·02 to 0·06)	0·02 (−0·02 to 0·05)	0·02 (−0·02 to 0·05)	0·00 (−0·01 to 0·01)	0·02 (−0·02 to 0·07)	0·05 (−0·05 to 0·13)
Severe infections and parasites*	0·13 (0·01 to 0·24)	0·08 (−0·07 to 0·20)	0 (−0·03 to 0·04)	0·09 (−0·02 to 0·18)	0·27 (0·05 to 0·44)	0·22 (0·05 to 0·37)
Antenatal and perinatal risk factors combined†	0·33 (0·21 to 0·43)	0·39 (0·22 to 0·52)	0·22 (0·10 to 0·32)	0·18 (0·10 to 0·25)	0·25 (0·15 to 0·33)	0·51 (0·36 to 0·63)

Data in parentheses are 95% CIs. Risk factors with negative population attributable fraction (cassava consumption, dogs in the household, and pork consumption) are excluded from the table. NA=not available.

* Combined population attributable fraction for admission to hospital with malaria or fever and positive for *T canis, T gondii, T solium*, or *O volvulus*.

† Combined population attributable fraction for abnormal antenatal period; home delivery; difficulties feeding, crying, or breathing; and any other problems after birth.

**Table 7:** Population attributable fractions associated with each risk factor in adults (aged ≥18 years)

	**All centres**	**Kilifi, Kenya**	**Agincourt, South Africa**	**Iganga-Mayuge, Uganda**	**Ifakara, Tanzania**	**Kintampo, Ghana**
Seizures in the family	0·12 (0·08 to 0·15)	0·10 (0·06 to 0·13)	0·07 (0·04 to 0·11)	0·18 (0·07 to 0·27)	0·17 (0·10 to 0·23)	0·14 (0·08 to 0·20)
Maternal seizures	0·01 (0 to 0·02)	0·02 (0 to 0·04)	0·00 (0 to 0·01)	0·02 (−0·03 to 0·07)	0·00 (0 to 0·01)	0·00 (NA)
Abnormal delivery	0·00 (−0·01 to 0·02)	0·00 (−0·01 to 0·02)	0·00 (−0·02 to 0·02)	0·00 (−0·01 to 0·02)	0·00 (−0·01 to 0·02)	0·01 (−0·02 to 0·03)
Home delivery	0·11 (−0·06 to 0·25)	0·13 (−0·07 to 0·28)	0·08 (−0·04 to 0·18)	0·07 (−0·04 to 0·16)	0·10 (−0·05 to 0·24)	0·13 (−0·07 to 0·29)
Problems after birth	0·06 (0·04 to 0·08)	0·04 (0·02 to 0·06)	0·04 (0·01 to 0·06)	0·03 (−0·03 to 0·08)	0·02 (0 to 0·04)	0·17 (0·10 to 0·23)
Head injury	0·02 (0 to 0·04)	0·03 (0·01 to 0·06)	0·01 (0 to 0·02)	0·00 (0 to 0)	0·01 (0 to 0·02)	0·04 (0 to 0·07)
Malnourished	0·03 (−0·01 to 0·07)	0·03 (−0·01 to 0·07)	0·03 (−0·01 to 0·07)	0·07 (−0·03 to 0·16)	0·03 (−0·01 to 0·07)	0·04 (−0·01 to 0·09)
Cats in household	0·01 (−0·11 to 0·12)	0·01 (−0·12 to 0·14)	0·00 (−0·01 to 0·02)	0·00 (0 to 0)	0·02 (−0·22 to 0·22)	0·01 (−0·09 to 0·10)
Eats cassava	0·26 (0·09 to 0·40)	0·27 (0·09 to 0·41)	0·24 (0·08 to 0·37)	0·32 (0·11 to 0·47)	0·21 (0·07 to 0·33)	0·31 (0·11 to 0·47)
Eats pork	0·04 (−0·05 to 0·11)	0·02 (−0·03 to 0·08)	0·02 (−0·03 to 0·07)	0·02 (−0·03 to 0·07)	0·06 (−0·09 to 0·20)	0·05 (−0·06 to 0·15)
Uses drugs	0·01 (−0·01 to 0·04)	0·02 (−0·02 to 0·06)	0·01 (−0·01 to 0·02)	0·04 (−0·05 to 0·12)	0·01 (−0·01 to 0·02)	0·01 (−0·01 to 0·03)
Hypertension	0·01 (0 to 0·03)	0·01 (0 to 0·02)	0·05 (−0·01 to 0·10)	0·00 (NA to NA)	0·00 (0 to 0·01)	0·00 (NA)
Stroke	0·01 (0 to 0·01)	0·00 (0 to 0·01)	0·02 (−0·01 to 0·05)	0·00 (0 to 0)	0·00 (0 to 0·01)	0·00 (0 to 0)
Diabetes mellitus	0·00 (0 to 0·01)	0·00 (NA)	0·02 (−0·01 to 0·04)	0·00 (NA)	0·00 (0 to 0·01)	0·00 (NA)
Positive for malaria IgG (schizont)	0·12 (−0·27 to 0·39)	0·13 (−0·30 to 0·42)	0·06 (−0·13 to 0·22)	0·14 (−0·33 to 0·45)	0·14 (−0·32 to 0·44)	0·14 (−0·33 to 0·45)
Admitted to hospital with malaria or fever, or both	0·02 (0 to 0·03)	0·03 (0 to 0·06)	0·00 (0 to 0)	0·02 (−0·02 to 0·05)	0·02 (0 to 0·05)	0·00 (NA)
Positive for *Toxocara canis* IgG4	0·16 (0·08 to 0·24)	0·21 (0·10 to 0·31)	0·04 (0·01 to 0·07)	0·14 (0·03 to 0·25)	0·35 (0·17 to 0·50)	0·11 (0·04 to 0·18)
Positive for *Toxoplasma gondii* IgG	0·13 (0·02 to 0·23)	0·13 (0·02 to 0·22)	0·04 (0 to 0·08)	0·11 (0 to 0·20)	0·15 (0·02 to 0·26)	0·24 (0·04 to 0·40)
Positive for *Taenia solium*	0·01 (0 to 0·02)	NA	NA	0·02 (−0·03 to 0·07)	0·02 (−0·01 to 0·05)	0·01 (−0·01 to 0·04)
Positive for *Onchocerca volvulus*	0·14 (0·08 to 0·20)	NA	NA	0·07 (−0·01 to 0·14)	0·30 (0·17 to 0·41)	0·29 (0·16 to 0·40)
Severe infections and parasites*	0·35 (0·24 to 0·44)	0·31 (0·16 to 0·44)	0·09 (0·04 to 0·14)	0·28 (0·12 to 0·40)	0·62 (0·44 to 0·74)	0·52 (0·34 to 0·65)

Data in parentheses are 95% CIs. Risk factors with negative population attributable fraction (alcohol consumption, dogs in the household, and HIV positive) are excluded from the table. NA=not available.

* Combined population attributable fraction for admission to hospital with malaria or fever and positive for *T canis, T gondii, T solium*, or *O volvulus.*

## Discussion

We have shown that the prevalence of active convulsive epilepsy varies between five large populations in sub-Saharan Africa (panel). The variation is unlikely to have been caused by differences in method, because we used the same screening methods, techniques, and definitions in all sites^24^, ^44^ to minimise the methodological component of heterogeneity reported in a meta-analysis of prevalence studies.^2^ The variation is probably a result of differences in risk factors. Risk factors related to the antenatal and perinatal periods were the most strongly associated with active convulsive epilepsy in children. Parasitic risk factors were strongly associated with active convulsive epilepsy in adults, but we recorded substantial variability between centres. Our multicentre study used well established health and demographic surveillance infrastructures, which allowed rapid and efficient screening of large populations.

Panel **Research in context**

**Systematic review**

We searched Medline for reports published between Jan 1, 1966, and Dec 31, 2012, and Cochrane reviews published at any time, with the terms “epilepsy”, “prevalence”, and “Africa”. We restricted searches to titles and abstracts, and to human populations. We used no language restrictions. We identified 65 reports, of which ten were relevant; we had reviewed seven of these in a previous report.^2^ The estimated prevalence of epilepsy varied, ranging between 4·0 and 29·5 cases per 1000 people. In a second review, we searched the same databases, with the same date restrictions and no language restrictions, with the search terms “epilepsy”, “(developing countries OR low and middle income countries OR LMIC)”, and “(case control OR risk factors OR causes OR etiology)”. We identified 62 reports (including 16 that were relevant) and four reviews.^3^, ^5^, ^42^, ^43^ Epilepsy had been previously associated with head injury, cerebrovascular disease, CNS infections and infestations (bacterial meningitis, viral encephalitis, malaria, neurocysticercosis, toxocariasis, and onchocerciasis), birth trauma, history of seizures, and family history of epilepsy.

**Interpretation**

We have shown that the prevalence of active convulsive epilepsy varies widely across sub-Saharan Africa and the variation is due to differences in the distribution of risk factors. Most active convulsive epilepsy in adults is attributable to parasitic infestations, but perinatal causes were important in children. Interventions to reduce exposure to parasites and improve perinatal care could substantially reduce the burden of epilepsy in this region.

The crude prevalence estimates from the three-stage method were lower than were those from the two-stage method in four centres. This difference could be partly attributed to the first stage of the three-stage method, which contributed more than 80% of false negatives in the validation study.^35^ False negatives in stage one were probably a result of stigma-related concealment of seizures, as was recorded in Kilifi,^35^ or possibly poor awareness of seizures in the family by key informants (ie, household heads).^45^

Our estimates confirm that prevalence of active convulsive epilepsy in sub-Saharan Africa is high and is within the range estimated for active epilepsy in countries of low and middle income,^2^ although previous estimates might include findings for non-convulsive epilepsies.^2^ Cross-sectional studies are thought to underestimate the prevalence of lifetime epilepsy by 75%, and non-convulsive epilepsy might constitute up to 50% of all epilepsies in population-based studies.^46^ Therefore, our estimates probably represent a quarter of all types of epilepsy in this region. The only other study of active convulsive epilepsy in this region (done in Kilifi) estimated a prevalence of 4·5 per 1000 people.^14^ This value was an underestimate, because the investigators did not adjust for the sensitivity of the entire three-stage method, although the crude prevalence was similar to that in our study. Our estimates varied by age and study centre, which reflects the difference in prevalence of risk factors.

Genetic susceptibility to epilepsy is well documented^47^, ^48^ and could explain the strong association with family history that we recorded. Family members could also share other non-genetic risk factors—eg, parasitic or perinatal risk factors. However, the effect of family history is potentially overestimated, because people with active convulsive epilepsy might be more aware of other family members who are affected by seizures than are individuals who do not have epilepsy.

Antenatal difficulties are associated with epilepsy.^49^ Birth asphyxia is a risk factor for epilepsy and neonatal mortality, but is preventable.^28^ In a 2005 review of simple interventions aimed at low-income countries, Darmstadt and colleagues^50^ showed that 43–73% of neonatal deaths in Africa could be prevented with implementation of a package of interventions to improve antenatal and perinatal care. The implementation of these interventions would probably affect the prevalence of epilepsy in this region.

Both malnutrition and cassava consumption were positively associated with epilepsy in adults in our study. These two risk factors are related; diets in which cassava is a staple are common in poor areas where malnutrition is widespread. Other epidemiological studies have also reported positive associations between epilepsy and malnutrition.^38^, ^51^ Biochemical mechanisms whereby malnutrition (particularly protein malnutrition) could lower the seizure threshold are known,^52^ but malnutrition is more likely to be a result of epilepsy than a cause. Additionally, parasitic infection could play a part as either a mediator or confounder of the relation between epilepsy and malnutrition.

The association between head injuries and active convulsive epilepsy was weaker than has been reported in other African studies.^14^, ^53^ Unlike our study, previous investigations might have involved assessment of head injuries before and after the first seizure, so the strength of these associations could be attributable to injuries occurring after the onset of epilepsy (ie, reverse causality), thereby overestimating the causal effect.^54^

Hypertension was positively associated with epilepsy in our study, as in European studies.^55^, ^56^ The prevalence of this risk factor was low in the communities that we studied, probably because hypertension is under-reported in sub-Saharan Africa.^57^ Hypertension might therefore have a much greater role than is suggested by our data, and could become important causes of epilepsy in the future, as a result of changes in lifestyle and the advancing epidemiological transition.

Exposure to the parasites *T gondii, T canis*, and *O volvulus* significantly increased the risk of epilepsy in adults. These associations are consistent with ecological studies^10^ and meta-analyses of case-control studies.^6^, ^12^, ^58^, ^59^ Ecological studies have shown that the prevalence of epilepsy is positively correlated with both the prevalence of toxoplasmosis seropositivity and onchocerciasis, and meta-analyses have further established positive associations for all three parasitic infections. In the nine case-control studies analysed for onchocerciasis,^58^ the ORs reported varied substantially and the overall association was of borderline significance (p=0·06). All three parasite species have been found in the CNS, but this location is uncommon for *T canis*^60^ and *O volvulus*;^61^ we cannot rule out the possibility that these associations have arisen through confounding (eg, poverty) or reverse causality (ie, people with epilepsy might have increased exposure to parasites).

The relation between cysticercosis and active convulsive epilepsy is well established.^6^, ^7^, ^8^ Worldwide, cysticercosis is thought to be the main risk factor for late-onset epilepsy and is an important risk factor for focal epilepsy in children.^5^, ^6^ We did not record an association between cysticercosis and active convulsive epilepsy in either adults or children, although we did note a strong association with adult-onset epilepsy. This finding might be explained by the low prevalence of the antibody to *T solium*, which will have lowered the statistical power to detect an association. By contrast, most African case-control studies that showed a significant association were done in settings where the prevalence of cysticercosis was much higher.^5^ Active convulsive epilepsy was not associated with HIV in our study, but individuals at risk of epilepsy because of an underlying condition caused by HIV will probably be selected against because of early mortality. Additionally, we did not record an association between exposure to malaria (as measured by antibodies to a schizont mixture) and active convulsive epilepsy, but were able to confirm that severe malaria is a cause of epilepsy, as has been previously reported.^4^

The estimated PAFs do not match the ORs, because they depend on the prevalence of the risk factor in individuals with the disorder as well as the OR. The cumulative PAF suggests that about 30% of paediatric epilepsy could be prevented with improvement of antenatal and perinatal care, and roughly 35% of adult epilepsy could be prevented with interruption of the transmission of parasites in sub-Saharan Africa.

We used a three-stage, cross-sectional screening method that had low sensitivity for identification of potential cases of active convulsive epilepsy. In Iganga-Mayuge, attrition between survey stages was high because of logistical difficulties with follow-up of eligible participants. However, we adjusted our estimates for these factors in our analyses. Additionally, the prevalence estimates from these studies might not be generalisable to many parts of sub-Saharan Africa, because the study sites were selected on the basis of endemicity of potential risk factors and availability of minimum resources necessary to support the studies.

The effect estimates from our study should be cautiously interpreted, particularly those that are susceptible to recall bias (eg, perinatal risk factors) and measurement error (eg, under-reporting of diabetes). We could only establish exposure status before the onset of epilepsy for two risk factors (admission to hospital for malaria or fever, and head injuries). For many risk factors—eg, malnutrition, alcohol, and parasitic infection—reverse causality is a potential source of bias. For parasitic risk factors, we used laboratory methods that establish exposure, but we could not detect direct parasite invasion of the CNS with techniques available in these sites. We adjusted for sociodemographic confounding factors, but residual confounding could remain—eg, individuals living in areas where parasites are common could be poorer and at higher risk of epilepsy than are those living in areas where parasites are less common. Additionally, we did not investigate some risk factors in this study (eg, brain tumours and other types of infection, particularly viral and bacterial)^3^ and some were only available for either adults or children (eg, data for perinatal events were obtained only for children).

In conclusion, the prevalence of active convulsive epilepsy varies across sub-Saharan Africa, and is related to the distribution and types of risk factors for epilepsy. Many cases of active convulsive epilepsy are attributable to parasitic disease, particularly in areas where onchocerciasis is common. Thus, parasitic-control programmes could reduce the prevalence of epilepsy. Furthermore, straightforward interventions aiming to improve antenatal and perinatal care could reduce the prevalence of epilepsy in sub-Saharan Africa.
