# Supplementary material for: Prevalence of active convulsive epilepsy in sub-Saharan Africa and associated risk factors: cross-sectional and case-control studies
Source: Lancet Neurol. 2013 Mar 13;12(3):253–63. doi: 10.1016/S1474-4422(13)70003-6 (PMC3581814; doi:10.1016/S1474-4422(13)70003-6)
Supplement: Supplementary appendix [file mmc1.pdf]

THE LANCET Neurology

Supplementary webappendix

This webappendix formed part of the original submission and has been peer reviewed. We post it as supplied by the authors.

Supplement to: Ngugi AK, Bottomley C, Kleinschmidt I, et al, for the SEEDS group. Prevalence of active convulsive epilepsy in sub-Saharan Africa and associated risk factors: cross-sectional and case-control studies. *Lancet Neurol* 2013; published online Jan 31. http://dx.doi.org/10.1016/S1474-4422(13)70003-6.

APPENDICES

Stage II (SII) screening questions

Q1. Have you ever had a fit?
Q2. Has someone ever told you that you have fits?
Q3. Have you ever been told that you have epilepsy or epileptic fits?
Q4. Have you ever had attacks in which you fall to the ground with loss of consciousness?
Q5. Have you ever fallen to the ground without a reason and experienced:

a) Twitching?

b) Shaking of the arms or legs without control?

c) Wetting yourself?

d) Biting of the tongue?

Q6. Have you ever been told by a doctor that you have epilepsy or epileptic fits?
If **Yes** to any of the above 6 questions:

I. When did the seizures start?
II. When was the last seizure?
III. Have you ever had any seizure within the last **5** years?
IV. If **Yes,** how many seizures have you had within the last one year?
V. Did **all** the seizures occur with a febrile illness?
VI. Are you currently using any drugs for convulsions?

Supplementary Table 1: Centre-specific comparison of the two- and three-stage methods for detecting cases of active convulsive epilepsy among participants selected in the population samples.

						Two-stage method (SII+SIII)		Three-stage method (SI+SII+SIII)		Comparison
**Study centre**	**Sample**	**Screened SI**	**Screened SII**	**Positive SII**	**Screened SIII**	**Cases detected (crude)**	***Adjusted prevalence (95% CI)**	**Cases detected (crude)**	***Adjusted prevalence (95% CI)**	**† Prevalence proportion (%) missed in 3-stages**
**Kilifi**	6000	5883	5594	68	38	23	9•3 (7•4-12•8)	17	7•0 (5•0-9•4)	24•7
**Agincourt**	4500	4209	3889	67	58	26	9•8 (6•9-13•4)	16	7•4 (5•0-10•4)	24•5
**Iganga**	5000	4436	4053	125	46	22	19•7 (15•7-24•5)	15	10•1 (7•4-13•6)	48•7
**Ifakara**	5000	4702	4985	54	49	31	9•8 (7•2-13•0)	22	15•3 (12•0-19•2)	Nil
**Kintampo**	5000	4183	4849	38	24	16	15•5 (12•2-19•4)	11	12•4 (9•3-16•3)	20•0
**Overall**	25 500	23 413	23 371	352	215	116		81		

*Adjusted for attrition and sensitivity of SII+SIII (76•7%) and SI+SII+SIII (48•6%); †Based on ratio of the two adjusted prevalence estimates.

Supplementary Table 2: Demographic characteristics of cases and controls at each centre (AGN=Agincourt, IFA=Ifakara, IGN=Iganga, KIL=Kilifi, KIN=Kintampo)

		**Agincourt**		**Ifakara**		**Iganga-Mayuge**		**Kilifi**		**Kintampo**		**All**	
		**% Control (N=261)**	**Case % (N=245)**	**% Control (N=625)**	**Case % (N=366)**	**% Control (N=239)**	**Case % (N=152)**	**% Control (N=527)**	**Case % (N=699)**	**% Control (N=381)**	**Case % (N=249)**	**% Control (N=2033)**	**Case % (N=1711)**
**Age†**	**0-5**	4	4	7	7	21	34	16	14	3	2	10	11
	**6-12**	16	11	28	20	29	29	23	23	19	13	24	20
	**13-18**	10	15	23	24	18	16	19	21	28	25	21	21
	**19-28**	20	19	15	18	18	13	16	20	28	38	19	21
	**29-49**	33	35	21	25	11	7	15	13	17	17	19	19
	**50+**	16	16	6	6	3	2	11	9	6	5	8	8
**Sex‡**	**Female**	68	47	49	52	54	47	53	49	51	45	54	49
	**Male**	32	53	51	48	46	53	47	51	49	55	46	51
**Education**	**None**	21	20	17	28	24	27	46	51	48	43	32	38
	**Primary**	36	40	58	55	44	50	44	40	5	4	40	39
	**Secondary and above**	41	37	3	1	23	16	7	3	45	51	19	16
	**Unknown**	2	3	23	16	9	7	3	5	3	3	10	7

† 4 individuals missing age, ‡18 individuals missing sex

Supplementary Table 3a: Risk factors for ACE in children by country (Agincourt)*

	Controls (n/N, %)	Cases (n/N, %)	Unadjusted OR (95% CI)	p value†	Adjusted OR (95% CI)	p value
**Seizures in the family**	6•9(5/72)	15•9(13/82)	2•52(0•79,9•50)	0•130	3•06(0•97,9•67)	0•057
**Maternal seizures**	0•0(0/72)	2•5(2/81)	NA	0•498	NA	NA
**Abnormal delivery**	10•6(7/66)	10•3(8/78)	0•96(0•29,3•32)	1	0•92(0•29,2•96)	0•892
**Abnormal antenatal period**	3•2(2/63)	5•7(4/70)	1•85(0•25,21•02)	0•683	1•13(0•18,7•02)	0•894
**Home delivery**	12•9(9/70)	16•5(13/79)	1•34(0•49,3•80)	0•646	1•51(0•56,4•11)	0•415
**Problems after birth**	1•4(1/71)	7•7(6/78)	5•83(0•67,272•03)	0•119	4•03(0•41,39•26)	0•230
**Difficulties feeding, crying or breathing**	4•5(3/66)	10•1(7/69)	2•37(0•51,14•76)	0•326	2•03(0•46,8•93)	0•348
**Head injury**	1•4(1/73)	7•3(6/82)	5•68(0•66,264•98)	0•121	4•36(0•48,39•80)	0•192
**Malnourished**	20•8(15/72)	19•7(13/66)	0•93(0•37,2•32)	1	0•87(0•35,2•15)	0•765
**Eats cassava**	50•7(37/73)	46•3(38/82)	0•84(0•43,1•66)	0•631	0•85(0•42,1•74)	0•664
**Dogs in household**	13•7(10/73)	25•6(21/82)	2•17(0•89,5•58)	0•073	1•93(0•80,4•62)	0•142
**Cats in household**	8•2(6/73)	4•9(4/82)	0•57(0•11,2•54)	0•518	0•54(0•13,2•26)	0•398
**Eats Pork**	15•1(11/73)	11•1(9/81)	0•70(0•24,2•01)	0•483	0•63(0•23,1•77)	0•385
**Malaria IgG +ve (schizont)**	22•0(13/59)	23•9(11/46)	1•11(0•40,3•06)	0•82	1•26(0•45,3•49)	0•662
**Hospitalised with malaria or fever**	0•0(0/73)	0•0(0/83)	NA	NA	NA	NA
**Toxocara canis IgG4 +ve**	22•0(13/59)	19•6(9/46)	0•86(0•29,2•46)	0•813	0•90(0•31,2•64)	0•854
**Toxoplasma gondii IgG +ve**	6•8(4/59)	2•2(1/46)	0•31(0•01,3•26)	0•383	0•57(0•05,6•20)	0•647
**Taenia solium +ve**	NA	NA	NA	NA	NA	NA
**Onchocerca volvulus +ve**	NA	NA	NA	NA	NA	NA
**HIV +ve**	12•1(7/58)	4•3(2/46)	0•33(0•03,1•88)	0•293	0•28(0•05,1•68)	0•163

* OR children adjusted for: age, sex, maternal education, maternal marital status and employment of either parent; † Fisher's exact test for unadjusted p-values
N=73 controls and N=83 cases, including 17 cases not identified as part of the three stage survey

Supplementary Table 3b: Risk factors for ACE in adults by country (Agincourt)*

			Unadjusted		Adjusted	
	Controls (n/N, %)	Cases (n/N, %)	OR (95% CI)	p value†	OR (95% CI)	p value
**Seizures in the family**	3•2(6/187)	13•7(28/204)	4•80(1•88,14•48)	0•0002	6•71(2•52,17•83)	0•0001
**Maternal seizures**	0•0(0/187)	0•5(1/204)	NA	1	NA	NA
**Abnormal delivery**	0•0(0/103)	6•0(11/184)	NA	0•009	NA	NA
**Home delivery**	45•2(47/104)	47•2(83/176)	1•08(0•65,1•81)	0•805	0•72(0•37,1•38)	0•319
**Problems after birth**	1•0(1/104)	4•4(8/182)	4•74(0•62,212•10)	0•163	9•05(1•00,81•98)	0•050
**Head injury**	1•6(3/188)	1•9(4/209)	1•20(0•20,8•32)	1	0•61(0•12,3•04)	0•545
**Drinks alcohol**	15•8(29/183)	16•3(33/203)	1•03(0•58,1•85)	1	0•75(0•40,1•40)	0•365
**Eats cassava**	62•0(116/187)	73•4(149/203)	1•69(1•08,2•66)	0•017	2•10(1•30,3•40)	0•002
**Eats Pork**	31•0(58/187)	22•7(46/203)	0•65(0•40,1•05)	0•067	0•65(0•40,1•08)	0•097
**Uses drugs**	3•3(6/183)	3•0(6/201)	0•91(0•24,3•46)	1	0•45(0•13,1•62)	0•222
**Hypertension**	7•0(13/187)	11•3(23/204)	1•70(0•80,3•77)	0•163	3•45(1•50,7•92)	0•004
**Stroke**	2•7(5/187)	4•9(10/204)	1•88(0•57,7•12)	0•299	2•18(0•65,7•26)	0•204
**Diabetes mellitus**	1•1(2/187)	3•4(7/204)	3•29(0•61,32•73)	0•178	3•28(0•61,17•59)	0•166
**Malnourished**	12•3(23/187)	14•4(26/180)	1•20(0•63,2•31)	0•646	0•92(0•47,1•77)	0•793
**Dogs in household**	21•9(41/187)	23•0(47/204)	1•07(0•64,1•77)	0•810	1•26(0•74,2•14)	0•387
**Cats in household**	8•6(16/186)	5•9(12/204)	0•66(0•28,1•55)	0•331	0•88(0•38,2•01)	0•758
**Malaria IgG +ve (schizont)**	37•5(57/152)	43•7(66/151)	1•29(0•80,2•10)	0•294	1•44(0•85,2•44)	0•180
**Hospitalised with malaria or fever**	0•0(0/188)	0•5(1/209)	NA	1	NA	NA
**Toxocara canis IgG4 +ve**	7•9(12/152)	9•9(15/151)	1•29(0•54,3•13)	0•553	1•61(0•64,4•02)	0•312
**Toxoplasma gondii IgG +ve**	11•2(17/152)	15•9(24/151)	1•50(0•73,3•12)	0•244	1•50(0•70,3•20)	0•293
**Taenia solium +ve**	NA	NA	NA	NA	NA	NA
**Onchocerca volvulus +ve**	NA	NA	NA	NA	NA	NA
**HIV +ve**	28•0(42/150)	22•5(34/151)	0•75(0•43,1•30)	0•291	0•69(0•37,1•28)	0•237

* OR adults adjusted for: age, sex, education, marital status and employment; † Fisher's exact test for unadjusted p-values
N=188 controls and N=209 cases, including 30 cases not identified as part of the three stage survey

Supplementary Table 4a: Risk factors for ACE in children by country (Ifakara) *

			Unadjusted		Adjusted	
	Controls (n/N, %)	Cases (n/N, %)	OR (95% CI)	p value†	OR (95% CI)	p value
**Seizures in the family**	10•5(36/343)	14•7(32/218)	1•47(0•85,2•52)	0•146	1•45(0•86,2•46)	0•161
**Maternal seizures**	1•2(4/344)	2•8(6/218)	2•41(0•56,11•71)	0•197	2•78(0•69,11•24)	0•152
**Abnormal delivery**	4•1(14/341)	3•2(7/217)	0•78(0•26,2•10)	0•655	0•76(0•30,1•94)	0•564
**Abnormal antenatal period**	2•3(8/341)	7•4(16/215)	3•35(1•32,9•18)	0•005	3•84(1•52,9•71)	0•004
**Home delivery**	36•7(121/330)	35•3(76/215)	0•94(0•65,1•37)	0•785	0•90(0•62,1•30)	0•557
**Problems after birth**	2•6(9/340)	8•8(19/216)	3•55(1•49,9•06)	0•002	3•73(1•59,8•76)	0•003
**Difficulties feeding, crying or breathing**	0•9(3/338)	10•3(22/213)	12•86(3•78,67•70)	<0•0001	13•29(3•79,46•62)	<0•0001
**Head injury**	0•6(2/341)	2•8(6/215)	4•87(0•86,49•59)	0•06	4•25(0•84,21•44)	0•079
**Malnourished**	15•5(50/322)	20•4(42/206)	1•39(0•86,2•25)	0•159	1•48(0•92,2•38)	0•108
**Eats cassava**	49•9(171/343)	57•3(125/218)	1•38(0•96,1•97)	0•069	1•23(0•87,1•75)	0•240
**Dogs in household**	84•3(290/344)	84•4(184/218)	1•01(0•62,1•66)	1	1•06(0•65,1•71)	0•826
**Cats in household**	87•7(300/342)	94•5(205/217)	2•39(1•20,5•11)	0•008	2•53(1•29,4•97)	0•007
**Eats Pork**	66•1(226/342)	59•6(130/218)	0•76(0•53,1•09)	0•127	0•73(0•51,1•05)	0•093
**Malaria IgG +ve (schizont)**	90•1(154/171)	95•6(153/160)	2•41(0•92,7•07)	0•058	1•71(0•63,4•61)	0•289
**Hospitalised with malaria or fever**	3•5(12/347)	8•2(18/220)	2•49(1•11,5•78)	0•020	2•74(1•23,6•10)	0•013
**Toxocara canis IgG4 +ve**	43•7(31/71)	55•1(43/78)	1•59(0•79,3•19)	0•191	1•60(0•78,3•28)	0•199
**Toxoplasma gondii IgG +ve**	31•3(50/160)	42•1(64/152)	1•60(0•98,2•62)	0•060	1•50(0•91,2•46)	0•111
**Taenia solium +ve**	0•0(0/172)	1•3(2/158)	NA	0•228	NA	NA
**Onchocerca volvulus +ve**	21•1(36/171)	21•3(34/160)	1•01(0•58,1•78)	1	1•04(0•60,1•81)	0•882
**HIV +ve**	8•2(14/171)	13•8(22/160)	1•79(0•84,3•93)	0•114	1•86(0•88,3•91)	0•102

* OR children adjusted for: age, sex, maternal education, maternal marital status and employment of either parent; † Fisher's exact test for unadjusted p-values
N=347 controls and N=220 cases, including 44 cases not identified as part of the three stage survey

Supplementary Table 4b: Risk factors for ACE in adults by country (Ifakara)*

			Unadjusted		Adjusted	
	Controls (n/N, %)	Cases (n/N, %)	OR (95% CI)	p value†	OR (95% CI)	p value
**Seizures in the family**	11•7(32/273)	28•4(64/225)	2•99(1•83,4•95)	<0•0001	3•20(1•91,5•37)	<0•0001
**Maternal seizures**	0•4(1/277)	2•2(5/230)	6•13(0•68,291•07)	0•096	5•28(0•56,50•17)	0•147
**Abnormal delivery**	6•8(19/278)	3•5(8/230)	0•49(0•18,1•20)	0•113	0•51(0•20,1•30)	0•158
**Home delivery**	55•9(124/222)	66•5(135/203)	1•57(1•04,2•37)	0•029	1•61(1•01,2•56)	0•044
**Problems after birth**	1•1(3/275)	2•6(6/229)	2•44(0•51,15•22)	0•312	2•09(0•48,9•07)	0•327
**Head injury**	0•4(1/278)	2•2(5/230)	6•16(0•68,292•12)	0•096	13•95(1•21,161•28)	0•035
**Drinks alcohol**	21•9(59/269)	19•2(41/214)	0•84(0•52,1•35)	0•498	1•08(0•63,1•83)	0•787
**Eats cassava**	52•9(147/278)	66•7(152/228)	1•78(1•22,2•60)	0•002	1•96(1•30,2•94)	0•001
**Eats Pork**	62•1(169/272)	70•9(163/230)	1•48(1•00,2•20)	0•047	1•52(1•00,2•32)	0•050
**Uses drugs**	5•1(14/272)	3•7(8/217)	0•71(0•25,1•84)	0•514	1•07(0•40,2•87)	0•898
**Hypertension**	0•0(0/274)	0•9(2/218)	NA	0•196	NA	NA
**Stroke**	0•0(0/273)	0•5(1/219)	NA	0•445	NA	NA
**Diabetes mellitus**	0•0(0/277)	0•5(1/219)	NA	0•442	NA	NA
**Malnourished**	10•2(28/274)	16•4(37/225)	1•73(0•99,3•04)	0•045	2•07(1•13,3•80)	0•019
**Dogs in household**	82•7(230/278)	81•7(187/229)	0•93(0•57,1•51)	0•816	1•06(0•64,1•76)	0•828
**Cats in household**	89•9(250/278)	90•4(207/229)	1•05(0•56,2•00)	0•882	1•35(0•70,2•63)	0•374
**Malaria IgG +ve (schizont)**	98•3(171/174)	97•7(173/177)	0•76(0•11,4•56)	1	0•86(0•15,4•97)	0•867
**Hospitalised with malaria or fever**	1•1(3/278)	3•9(9/230)	3•73(0•92,21•64)	0•043	3•51(0•84,14•59)	0•085
**Toxocara canis IgG4 +ve**	58•5(48/82)	79•4(85/107)	2•74(1•37,5•49)	0•002	2•43(1•16,5•08)	0•018
**Toxoplasma gondii IgG +ve**	53•4(87/163)	56•1(92/164)	1•12(0•71,1•77)	0•657	1•10(0•66,1•86)	0•710
**Taenia solium +ve**	0•6(1/174)	3•4(6/176)	6•11(0•73,282•40)	0•121	10•12(1•13,90•89)	0•039
**Onchocerca volvulus +ve**	37•9(66/174)	54•2(96/177)	1•94(1•24,3•04)	0•003	1•87(1•15,3•05)	0•012
**HIV +ve**	17•2(30/174)	20•9(37/177)	1•27(0•72,2•25)	0•417	1•12(0•61,2•05)	0•716

*OR adults adjusted for: age, sex, education, marital status and employment; † Fisher's exact test for unadjusted p-values
N=278 controls and N=230 cases, including 40 cases not identified as part of the three stage survey

Supplementary Table 5a: Risk factors for ACE in children by country (Iganga-Mayuge) *

			Unadjusted		Adjusted	
	**Controls (n/N, %)**	**Cases (n/N, %)**	**OR (95% CI)**	**p value†**	**OR (95% CI)**	**p value**
**Seizures in the family**	8•4(13/154)	23•1(40/173)	3•26(1•62,6•93)	0•0003	2•99(1•51,5•91)	0•002
**Maternal seizures**	0•6(1/155)	2•9(5/170)	4•67(0•51,222•09)	0•217	3•93(0•42,36•94)	0•231
**Abnormal delivery**	1•3(2/150)	3•5(6/172)	2•67(0•47,27•41)	0•292	3•60(0•69,18•91)	0•129
**Abnormal antenatal period**	1•3(2/151)	8•2(14/171)	6•64(1•48,60•93)	0•004	15•45(1•98,120•67)	0•009
**Home delivery**	12•4(19/153)	28•9(50/173)	2•87(1•55,5•43)	0•0004	2•90(1•55,5•43)	0•0009
**Problems after birth**	2•6(4/152)	5•8(10/172)	2•28(0•64,10•17)	0•182	2•98(0•88,10•07)	0•078
**Difficulties feeding, crying or breathing**	0•7(1/151)	6•6(11/166)	10•65(1•51,461•11)	0•006	11•12(1•39,88•86)	0•023
**Head injury**	0•7(1/153)	3•5(6/170)	5•56(0•66,257•40)	0•125	3•78(0•42,34•03)	0•236
**Malnourished**	23•7(31/131)	21•3(32/150)	0•87(0•48,1•60)	0•669	0•82(0•45,1•49)	0•507
**Eats cassava**	91•0(141/155)	92•5(160/173)	1•22(0•51,2•93)	0•689	1•19(0•48,2•91)	0•707
**Dogs in household**	16•1(25/155)	6•9(12/173)	0•39(0•17,0•84)	0•014	0•42(0•20,0•88)	0•022
**Cats in household**	3•9(6/155)	1•7(3/173)	0•44(0•07,2•10)	0•316	0•46(0•11,1•98)	0•298
**Eats Pork**	10•4(16/154)	12•9(22/171)	1•27(0•61,2•71)	0•605	1•28(0•60,2•73)	0•515
**Malaria IgG +ve (schizont)**	99•2(125/126)	100•0(91/91)	NA	1	NA	NA
**Hospitalised with malaria or fever**	0•6(1/155)	0•0(0/173)	NA	0•473	NA	NA
**Toxocara canis IgG4 +ve**	18•3(23/126)	18•7(17/91)	1•03(0•48,2•17)	1	1•18(0•55,2•57)	0•669
**Toxoplasma gondii IgG +ve**	19•8(25/126)	22•0(20/91)	1•14(0•55,2•32)	0•736	0•92(0•44,1•92)	0•831
**Taenia solium +ve**	1•6(2/126)	1•1(1/91)	0•69(0•01,13•44)	1	1•50(0•07,33•68)	0•798
**Onchocerca volvulus +ve**	4•8(6/126)	12•1(11/91)	2•75(0•89,9•39)	0•071	2•93(1•00,8•57)	0•049
**HIV +ve**	2•4(3/126)	3•3(3/91)	1•40(0•18,10•66)	0•697	1•06(0•17,6•79)	0•950

* OR children adjusted for: age, sex, maternal education, maternal marital status and employment of either parent; † Fisher's exact test for unadjusted p-values
N=155 controls and N=173 cases, including 55 cases not identified as part of the three stage survey

Supplementary Table 5b: Risk factors for ACE in adults by country (Iganga-Mayuge)*

			Unadjusted		Adjusted	
	Controls (n/N, %)	Cases (n/N, %)	OR (95% CI)	p value†	OR (95% CI)	p value
**Seizures in the family**	10•8(9/83)	32•8(20/61)	4•01(1•56,10•87)	0•002	5•19(1•49,18•16)	0•010
**Maternal seizures**	0•0(0/84)	1•6(1/61)	NA	0•421	NA	NA
**Abnormal delivery**	2•4(2/83)	1•7(1/60)	0•69(0•01,13•51)	1	1•67(0•08,35•63)	0•741
**Home delivery**	42•7(35/82)	31•1(19/61)	0•61(0•28,1•29)	0•168	0•49(0•16,1•48)	0•207
**Problems after birth**	0•0(0/79)	7•3(4/55)	NA	0•027	NA	NA
**Head injury**	0•0(0/84)	0•0(0/62)	NA	NA	NA	NA
**Drinks alcohol**	11•6(8/69)	15•4(8/52)	1•39(0•42,4•59)	0•595	3•21(0•69,15•01)	0•138
**Eats cassava**	92•8(77/83)	100•0(61/61)	•(1•21,•)	0•039	1•00(1•00,1•00)	
**Eats Pork**	11•0(9/82)	19•4(12/62)	1•95(0•69,5•63)	0•233	1•55(0•41,5•84)	0•519
**Uses drugs**	30•0(21/70)	20•4(11/54)	0•60(0•23,1•48)	0•301	0•66(0•22,1•93)	0•449
**Hypertension**	1•2(1/83)	1•6(1/62)	1•34(0•02,106•78)	1	NA	NA
**Stroke**	0•0(0/83)	1•6(1/62)	NA	0•428	NA	NA
**Diabetes mellitus**	0•0(0/83)	0•0(0/62)	NA	NA	NA	NA
**Malnourished**	12•3(9/73)	31•1(19/61)	3•22(1•23,8•81)	0•01	2•41(0•70,8•32)	0•163
**Dogs in household**	13•4(11/82)	9•7(6/62)	0•69(0•20,2•19)	0•606	1•68(0•34,8•17)	0•522
**Cats in household**	2•4(2/82)	1•6(1/62)	0•66(0•01,12•90)	1	4•45(0•28,70•53)	0•29
**Malaria IgG +ve (schizont)**	100•0(73/73)	100•0(40/40)	NA	NA	NA	NA
**Hospitalised with malaria or fever**	0•0(0/84)	1•6(1/62)	NA	0•425	NA	NA
**Toxocara canis IgG4 +ve**	35•6(26/73)	45•0(18/40)	1•48(0•62,3•49)	0•420	1•77(0•58,5•41)	0•315
**Toxoplasma gondii IgG +ve**	41•1(30/73)	35•0(14/40)	0•77(0•32,1•84)	0•552	0•99(0•31,3•13)	0•985
**Taenia solium +ve**	0•0(0/73)	2•5(1/40)	NA	0•354	NA	NA
**Onchocerca volvulus +ve**	8•2(6/73)	5•0(2/40)	0•59(0•06,3•51)	0•710	0•32(0•03,3•98)	0•377
**HIV +ve**	2•7(2/73)	5•0(2/40)	1•87(0•13,26•56)	0•614	57•15(2•28,1431•47)	0•014

* OR adults adjusted for: age, sex, education, marital status and employment; † Fisher's exact test for unadjusted p-values
N=84 controls and N=62 cases, including 28 cases not identified as part of the three stage survey

Supplementary Table 6a: Risk factors for ACE in children by country (Kilifi)*

			Unadjusted		Adjusted	
	Controls (n/N, %)	Cases (n/N, %)	OR (95% CI)	p value†	OR (95% CI)	p value
**Seizures in the family**	15•6(45/288)	16•7(70/418)	1•09(0•71,1•68)	0•756	1•00(0•65,1•53)	0•992
**Maternal seizures**	1•0(3/289)	2•4(10/418)	2•34(0•59,13•31)	0•258	1•88(0•50,7•05)	0•349
**Abnormal delivery**	3•5(10/286)	6•0(25/417)	1•76(0•80,4•17)	0•159	1•90(0•86,4•23)	0•115
**Abnormal antenatal period**	12•3(35/285)	21•3(87/408)	1•94(1•24,3•06)	0•002	1•88(1•20,2•93)	0•006
**Home delivery**	84•4(243/288)	84•2(352/418)	0•99(0•64,1•52)	1	0•89(0•56,1•40)	0•609
**Problems after birth**	5•9(17/288)	13•8(57/414)	2•55(1•42,4•77)	0•0007	2•15(1•20,3•86)	0•010
**Difficulties feeding, crying or breathing**	2•8(8/285)	8•3(34/408)	3•15(1•40,7•98)	0•003	2•60(1•17,5•79)	0•019
**Head injury**	3•5(10/288)	8•6(36/419)	2•61(1•24,6•00)	0•008	2•44(1•14,5•21)	0•021
**Malnourished**	24•5(67/274)	25•5(100/392)	1•06(0•73,1•54)	0•786	1•11(0•76,1•63)	0•597
**Eats cassava**	80•3(232/289)	80•9(338/418)	1•04(0•70,1•54)	0•847	0•92(0•60,1•41)	0•707
**Dogs in household**	36•5(105/288)	33•8(142/420)	0•89(0•64,1•23)	0•471	0•90(0•65,1•26)	0•548
**Cats in household**	46•5(134/288)	54•8(229/418)	1•39(1•02,1•90)	0•032	1•37(0•99,1•89)	0•054
**Eats Pork**	16•1(46/285)	17•4(71/409)	1•09(0•71,1•68)	0•757	1•00(0•65,1•53)	0•990
**Malaria IgG +ve (schizont)**	60•6(83/137)	59•7(92/154)	0•97(0•59,1•59)	0•905	0•86(0•50,1•46)	0•574
**Hospitalised with malaria or fever**	4•2(12/289)	9•0(38/423)	2•28(1•14,4•87)	0•016	2•19(1•09,4•41)	0•028
**Toxocara canis IgG4 +ve**	29•2(40/137)	33•1(51/154)	1•20(0•71,2•04)	0•527	1•27(0•75,2•17)	0•375
**Toxoplasma gondii IgG +ve**	15•3(21/137)	22•1(34/154)	1•57(0•83,3•01)	0•177	1•74(0•90,3•39)	0•101
**Taenia solium +ve**	NA	NA	NA	NA	NA	NA
**Onchocerca volvulus +ve**	NA	NA	NA	NA	NA	NA
**HIV +ve**	1•5(2/134)	6•0(9/150)	4•21(0•85,40•59)	0•065	4•30(0•82,22•43)	0•084

* OR children adjusted for: age, sex, maternal education, maternal marital status and employment of either parent; † Fisher's exact test for unadjusted p-values
N=289 controls and N=423 case, including 40 cases not identified as part of the three stage survey

Supplementary Table 6b: Risk factors for ACE in adults by country (Kilifi)*

			Unadjusted		Adjusted	
	Controls (n/N, %)	Cases (n/N, %)	OR (95% CI)	p value†	OR (95% CI)	p value
**Seizures in the family**	13•5(32/237)	17•2(59/343)	1•33(0•82,2•20)	0•247	1•25(0•76,2•06)	0•384
**Maternal seizures**	0•8(2/237)	2•9(10/343)	3•53(0•74,33•35)	0•135	3•02(0•61,14•86)	0•174
**Abnormal delivery**	0•0(0/236)	3•0(10/337)	NA	0•007	NA	NA
**Home delivery**	83•2(198/238)	81•2(281/346)	0•87(0•55,1•38)	0•584	1•01(0•62,1•65)	0•969
**Problems after birth**	1•3(3/237)	4•8(16/332)	3•95(1•11,21•35)	0•03	3•00(0•77,11•64)	0•113
**Head injury**	2•5(6/238)	5•8(20/346)	2•37(0•90,7•32)	0•068	2•57(0•94,7•02)	0•066
**Drinks alcohol**	23•6(55/233)	12•9(44/342)	0•48(0•30,0•76)	0•001	0•55(0•33,0•92)	0•023
**Eats cassava**	91•9(217/236)	84•3(291/345)	0•47(0•26,0•84)	0•007	0•43(0•24,0•80)	0•008
**Eats Pork**	34•2(81/237)	26•8(92/343)	0•71(0•49,1•03)	0•065	0•74(0•50,1•10)	0•138
**Uses drugs**	12•1(28/232)	11•7(40/342)	0•96(0•56,1•68)	0•896	1•44(0•81,2•55)	0•217
**Hypertension**	0•8(2/237)	2•3(8/344)	2•80(0•55,27•23)	0•212	4•61(0•91,23•47)	0•065
**Stroke**	0•0(0/237)	0•6(2/345)	NA	0•516	NA	NA
**Diabetes mellitus**	0•0(0/237)	0•0(0/346)	NA	NA	NA	NA
**Malnourished**	14•2(33/232)	15•8(54/341)	1•13(0•69,1•88)	0•637	1•02(0•61,1•71)	0•947
**Dogs in household**	35•3(84/238)	27•2(94/345)	0•69(0•47,1•00)	0•044	0•69(0•47,1•02)	0•061
**Cats in household**	52•9(126/238)	53•2(184/346)	1•01(0•72,1•42)	1	0•96(0•67,1•37)	0•804
**Malaria IgG +ve (schizont)**	93•0(120/129)	93•2(136/146)	1•02(0•35,2•90)	1	1•27(0•44,3•62)	0•659
**Hospitalised with malaria or fever**	2•9(7/238)	5•5(19/346)	1•92(0•76,5•48)	0•158	1•95(0•74,5•14)	0•177
**Toxocara canis IgG4 +ve**	29•5(38/129)	49•3(72/146)	2•33(1•38,3•96)	0•0009	1•76(1•01,3•06)	0•044
**Toxoplasma gondii IgG +ve**	42•6(55/129)	45•9(67/146)	1•14(0•69,1•89)	0•628	1•40(0•82,2•40)	0•218
**Taenia solium +ve**	NA	NA	NA	NA	NA	NA
**Onchocerca volvulus +ve**	NA	NA	NA	NA	NA	NA
**HIV +ve**	3•2(4/126)	7•8(11/141)	2•58(0•74,11•37)	0•117	3•51(1•01,12•25)	0•049

*OR adults adjusted for: age, sex, education, marital status and employment; † Fisher's exact test for unadjusted p-values
N=238 controls N=346 cases, including 30 cases not identified as part of the three stage survey

Supplementary Table 7a: Risk factors for ACE in children by country (Kintampo) *

			Unadjusted		Adjusted	
	Controls (n/N, %)	Cases (n/N, %)	OR (95% CI)	p value†	OR (95% CI)	p value
**Seizures in the family**	14•0(24/172)	30•8(44/143)	2•74(1•52,5•02)	0•0003	3•31(1•83,5•96)	<0•0001
**Maternal seizures**	0•6(1/172)	1•4(2/144)	2•41(0•12,142•89)	0•593	2•92(0•26,32•81)	0•386
**Abnormal delivery**	3•7(6/164)	9•2(13/141)	2•67(0•91,8•80)	0•057	2•99(1•07,8•34)	0•036
**Abnormal antenatal period**	11•5(19/165)	17•8(24/135)	1•66(0•83,3•38)	0•138	1•87(0•94,3•75)	0•075
**Home delivery**	71•3(119/167)	79•6(113/142)	1•57(0•90,2•77)	0•113	1•47(0•85,2•55)	0•173
**Problems after birth**	2•4(4/164)	7•7(11/142)	3•36(0•96,14•75)	0•036	3•51(1•02,12•06)	0•047
**Difficulties feeding, crying or breathing**	0•6(1/164)	31•6(43/136)	75•37(12•27,3062•68)	<0•0001	73•04(9•81,544•07)	<0•0001
**Head injury**	18•7(32/171)	25•0(36/144)	1•45(0•82,2•57)	0•216	1•45(0•82,2•54)	0•200
**Malnourished**	20•8(32/154)	12•5(15/120)	0•54(0•26,1•11)	0•077	0•56(0•28,1•12)	0•102
**Eats cassava**	100•0(170/170)	96•5(138/143)	0•00(0•00,0•63)	0•019	1•00(1•00,1•00)	
**Dogs in household**	48•3(83/172)	47•6(68/143)	0•97(0•61,1•55)	0•910	0•93(0•58,1•50)	0•777
**Cats in household**	33•1(57/172)	34•3(49/143)	1•05(0•64,1•73)	0•905	0•97(0•59,1•58)	0•896
**Eats Pork**	51•2(88/172)	53•8(77/143)	1•11(0•70,1•78)	0•652	1•12(0•70,1•78)	0•646
**Malaria IgG +ve (schizont)**	99•1(116/117)	100•0(93/93)	NA	1	NA	NA
**Hospitalised with malaria or fever**	0•0(0/173)	0•0(0/145)	NA	NA	NA	NA
**Toxocara canis IgG4 +ve**	13•7(16/117)	14•0(13/93)	1•03(0•43,2•42)	1	0•97(0•42,2•24)	0•935
**Toxoplasma gondii IgG +ve**	42•7(50/117)	51•6(48/93)	1•43(0•80,2•56)	0•213	1•06(0•58,1•94)	0•852
**Taenia solium +ve**	6•9(8/116)	4•3(4/93)	0•61(0•13,2•36)	0•554	0•40(0•10,1•66)	0•207
**Onchocerca volvulus +ve**	16•2(19/117)	33•3(31/93)	2•58(1•28,5•26)	0•005	2•32(1•12,4•78)	0•023
**HIV +ve**	20•5(24/117)	25•8(24/93)	1•35(0•67,2•71)	0•410	1•35(0•67,2•73)	0•395

* OR children adjusted for: age, sex, maternal education, maternal marital status and employment of either parent; † Fisher's exact test for unadjusted p-values
N=173 controls and N=145 cases, including 57 cases not identified as part of the three stage survey

Supplementary Table 7b: Risk factors for ACE in adults by country (Kintampo)*

			Unadjusted		Adjusted	
	**Controls (n/N, %)**	**Cases (n/N, %)**	**OR (95% CI)**	**p value†**	**OR (95% CI)**	**p value**
**Seizures in the family**	13•0(27/207)	24•8(60/242)	2•20(1•30,3•77)	0•002	1•83(1•05,3•20)	0•034
**Maternal seizures**	0•0(0/207)	0•4(1/243)	NA	1	NA	NA
**Abnormal delivery**	8•1(16/198)	6•7(16/239)	0•82(0•37,1•80)	0•586	0•82(0•36,1•90)	0•646
**Home delivery**	85•6(167/195)	86•0(208/242)	1•03(0•57,1•82)	1	1•07(0•58,1•99)	0•824
**Problems after birth**	3•1(6/196)	18•3(44/240)	7•11(2•92,20•82)	<0•0001	8•74(3•21,23•83)	<0•0001
**Head injury**	4•3(9/208)	6•1(15/245)	1•44(0•58,3•82)	0•529	1•26(0•49,3•21)	0•633
**Drinks alcohol**	24•1(49/203)	27•3(66/242)	1•18(0•75,1•85)	0•514	1•51(0•91,2•52)	0•111
**Eats cassava**	93•2(193/207)	98•0(240/245)	3•48(1•16,12•54)	0•017	3•92(1•14,13•54)	0•031
**Eats Pork**	41•3(86/208)	49•0(119/243)	1•36(0•92,2•01)	0•108	1•68(1•09,2•58)	0•018
**Uses drugs**	1•9(4/206)	4•5(11/242)	2•40(0•70,10•50)	0•187	2•80(0•78,10•14)	0•116
**Hypertension**	2•5(5/202)	0•0(0/243)	0•00(0•00,0•63)	0•019	NA	NA
**Stroke**	0•0(0/202)	0•0(0/243)	NA	NA	NA	NA
**Diabetes mellitus**	0•5(1/201)	0•0(0/243)	0•00(0•00,•)	0•453	NA	NA
**Malnourished**	17•5(34/194)	18•6(41/221)	1•07(0•63,1•83)	0•800	0•97(0•53,1•77)	0•923
**Dogs in household**	49•5(103/208)	43•9(107/244)	0•80(0•54,1•17)	0•256	0•90(0•59,1•37)	0•616
**Cats in household**	30•3(63/208)	36•1(88/244)	1•30(0•86,1•97)	0•230	1•28(0•82,2•00)	0•275
**Malaria IgG +ve (schizont)**	100•0(175/175)	99•5(187/188)	0•00(0•00,•)	1	NA	NA
**Hospitalised with malaria or fever**	0•0(0/208)	0•0(0/245)	NA	NA	NA	NA
**Toxocara canis IgG4 +ve**	20•6(36/175)	24•5(46/188)	1•25(0•74,2•12)	0•383	1•26(0•72,2•21)	0•411
**Toxoplasma gondii IgG +ve**	67•4(118/175)	81•9(154/188)	2•19(1•31,3•68)	0•002	1•99(1•15,3•45)	0•014
**Taenia solium +ve**	4•0(7/174)	3•7(7/188)	0•92(0•27,3•15)	1	0•83(0•24,2•93)	0•774
**Onchocerca volvulus +ve**	32•0(56/175)	50•5(95/188)	2•17(1•39,3•41)	0•0004	2•09(1•29,3•40)	0•003
**HIV +ve**	30•9(54/175)	29•3(55/188)	0•93(0•58,1•49)	0•819	0•76(0•45,1•28)	0•303

*OR adults adjusted for: age, sex, education, marital status and employment; † Fisher's exact test for unadjusted p-values
N=208 controls and N=245 cases, including 84 cases not identified as part of the three stage survey

Supplementary Table 8: Prevalence of risk factors among children by country

	Agincourt		Ifakara		Iganga-Mayuge		Kilifi		Kintampo	
	% Control (N=261)	% Case (N=245)	% Control (N=625)	% Case (N=366)	% Control (N=239)	% Case (N=152)	% Control (N=527)	% Case (N=699)	% Control (N=381)	% Case (N=249)
**Seizures in the family**	7	14	10	15	8	22	16	16	14	33
**Maternal seizures**	0	3	1	3	1	3	1	2	1	2
**Abnormal delivery**	11	10	4	3	1	3	3	6	4	9
**Abnormal antenatal period**	3	8	2	7	1	9	12	21	12	15
**Home delivery**	13	20	36	38	12	25	84	85	71	78
**Problems after birth**	1	8	3	9	3	9	6	13	2	6
**Difficulties feeding, crying or breathing**	5	13	1	12	1	6	3	8	1	39
**Head injury**	1	3	1	3	1	4	3	9	19	23
**Malnourished**	21	13	15	20	24	23	24	26	21	12
**Eats cassava**	51	44	50	59	91	91	80	80	100	97
**Dogs in household**	14	26	84	84	16	7	36	34	48	45
**Cats in household**	8	0	88	95	4	1	47	54	33	36
**Eats Pork**	15	12	66	58	10	16	16	18	51	57
**Malaria IgG +ve (schizont)**	22	24	90	97	99	100	61	60	99	100
**Hospitalised with malaria or fever**	0	0	3	6	0	0	4	9	0	0
**Toxocara canis IgG4 +ve**	22	13	44	56	18	16	29	34	14	14
**Toxoplasma gondii IgG +ve**	7	0	31	43	20	19	15	20	43	42
**Taenia solium +ve**	NA	NA	0	2	2	2	NA	NA	7	4
**Onchocerca volvulus +ve**	NA	NA	21	22	5	12	NA	NA	16	33
**HIV +ve**	12	8	8	11	2	2	1	7	21	21

Supplementary Table 9: Prevalence of risk factors among adults by country

	Agincourt		Ifakara		Iganga-Mayuge		Kilifi		Kintampo	
	% Control (N=261)	% Case (N=245)	% Control (N=625)	% Case (N=366)	% Control (N=239)	% Case (N=152)	% Control (N=527)	% Case (N=699)	% Control (N=381)	% Case (N=249)
**Seizures in the family**	3	13	12	31	11	28	14	17	13	25
**Maternal seizures**	0	1	0	1	0	3	1	3	0	0
**Abnormal delivery**	0	4	7	3	3	3	0	3	8	6
**Home delivery**	45	50	57	67	44	42	83	81	86	85
**Problems after birth**	1	4	1	3	0	3	1	5	3	20
**Head injury**	2	1	0	2	0	0	2	6	5	7
**Drinks alcohol**	16	14	23	22	12	10	24	12	24	27
**Eats cassava**	62	75	53	66	93	100	92	85	93	98
**Eats Pork**	31	24	63	71	11	24	34	27	41	49
**Uses drugs**	3	3	5	3	31	23	12	12	2	5
**Hypertension**	7	11	0	1	1	0	1	2	2	0
**Stroke**	3	4	0	1	0	0	0	1	0	0
**Diabetes mellitus**	1	3	0	1	0	0	0	0	0	0
**Malnourished**	12	15	10	16	13	36	14	15	18	19
**Dogs in household**	22	24	83	81	14	6	35	28	50	43
**Cats in household**	9	6	90	88	3	0	53	53	30	38
**Malaria IgG +ve (schizont)**	38	45	98	98	100	100	93	93	100	100
**Hospitalised with malaria or fever**	1	0	1	3	0	3	3	6	0	0
**Toxocara canis IgG4 +ve**	8	9	62	84	34	38	29	50	21	25
**Toxoplasma gondii IgG +ve**	11	15	54	56	43	38	43	44	67	84
**Taenia solium +ve**	NA	NA	1	4	0	4	NA	NA	4	3
**Onchocerca volvulus +ve**	NA	NA	38	56	9	12	NA	NA	32	52
**HIV +ve**	28	22	18	21	3	4	3	5	31	28

Supplementary Table 10: Risk factors for focal epilepsy in adults

	Controls (n/N, %)	Cases (n/N, %)	Odds ratio (95% CI)*	p value
**Seizures in the family**	10•7(106/987)	22•6(107/473)	2•70(1•94,3•74)	<0•0001
**Maternal seizures**	0•3(3/992)	1•1(5/475)	2•74(0•56,13•46)	0•213
**Abnormal delivery**	4•1(37/898)	3•2(15/465)	1•02(0•51,2•01)	0•959
**Home delivery**	67•9(571/841)	70•1(312/445)	1•17(0•85,1•60)	0•330
**Problems after birth**	1•5(13/891)	6•3(29/460)	5•97(2•82,12•65)	<0•0001
**Head injury**	1•8(18/996)	3•1(15/478)	2•07(0•97,4•41)	0•060
**Drinks alcohol**	20•9(200/957)	17•0(79/464)	0•90(0•64,1•25)	0•518
**Eats cassava**	75•7(750/991)	80•0(380/475)	1•62(1•18,2•21)	0•002
**Eats Pork**	40•9(403/986)	40•2(191/475)	1•00(0•77,1•29)	0•977
**Uses drugs**	7•6(73/963)	5•6(26/461)	0•99(0•58,1•67)	0•958
**Hypertension**	2•1(21/983)	3•6(17/470)	1•58(0•75,3•30)	0•227
**Stroke**	0•5(5/982)	1•3(6/472)	1•75(0•49,6•29)	0•393
**Diabetes mellitus**	0•3(3/985)	1•1(5/473)	3•26(0•69,15•43)	0•135
**Malnourished**	13•2(127/960)	18•0(82/456)	1•46(1•04,2•04)	0•027
**Dogs in household**	47•2(469/993)	39•6(188/475)	0•81(0•61,1•06)	0•130
**Cats in household**	46•1(457/992)	45•4(216/476)	1•01(0•74,1•38)	0•943
**Malaria IgG +ve (schizont)**	84•8(596/703)	79•3(264/333)	1•16(0•71,1•91)	0•550
**Hospitalised with malaria or fever**	1•1(11/996)	2•5(12/478)	1•91(0•79,4•61)	0•152
**Toxocara canis IgG4 +ve**	26•2(160/611)	34•1(101/296)	1•63(1•10,2•41)	0•015
**Toxoplasma gondii IgG +ve**	44•4(307/692)	45•4(149/328)	1•55(1•10,2•17)	0•011
**Taenia solium +ve**	1•9(8/421)	5•3(8/152)	3•80(1•28,11•23)	0•016
**Onchocerca volvulus +ve**	30•3(128/422)	57•1(88/154)	2•81(1•81,4•37)	<0•0001
**HIV +ve**	18•9(132/698)	19•9(66/332)	0•93(0•63,1•36)	0•691

*OR adjusted for age, sex, education, marital status, employment and country

Supplementary Table 11: Risk factors for focal epilepsy in children

	Controls (n/N, %)	Cases (n/N, %)	Odds ratio (95% CI)*	p value
**Seizures in the family**	11•9(122/1028)	17•0(62/364)	1•62(1•13,2•33)	0•008
**Maternal seizures**	0•9(9/1031)	1•9(7/360)	1•88(0•63,5•61)	0•256
**Abnormal delivery**	3•9(39/1006)	7•0(25/357)	1•99(1•14,3•47)	0•015
**Abnormal antenatal period**	6•6(66/1004)	11•6(40/344)	1•64(1•03,2•61)	0•036
**Home delivery**	50•6(510/1007)	58•9(211/358)	1•07(0•78,1•47)	0•671
**Problems after birth**	3•5(35/1014)	9•5(34/358)	2•47(1•46,4•16)	0•0007
**Difficulties feeding, crying or breathing**	1•6(16/1003)	10•4(36/346)	5•78(3•04,10•97)	<0•0001
**Head injury**	4•5(46/1025)	7•4(27/363)	2•48(1•38,4•45)	0•002
**Malnourished**	20•4(194/952)	19•0(63/332)	0•98(0•70,1•38)	0•905
**Eats cassava**	73•0(751/1029)	70•2(254/362)	0•98(0•72,1•33)	0•895
**Dogs in household**	49•7(512/1031)	47•4(172/363)	1•00(0•74,1•34)	0•974
**Cats in household**	48•8(502/1029)	55•7(201/361)	1•28(0•93,1•78)	0•131
**Eats Pork**	37•8(387/1025)	31•3(111/355)	0•88(0•64,1•20)	0•415
**Malaria IgG +ve (schizont)**	80•5(491/610)	69•5(132/190)	0•79(0•48,1•32)	0•369
**Hospitalised with malaria or fever**	2•3(24/1036)	6•3(23/365)	2•11(1•13,3•95)	0•020
**Toxocara canis IgG4 +ve**	24•1(123/510)	34•4(53/154)	1•23(0•79,1•91)	0•365
**Toxoplasma gondii IgG +ve**	25•0(150/599)	27•6(51/185)	1•25(0•81,1•92)	0•323
**Taenia solium +ve**	2•4(10/414)	2•0(2/98)	1•97(0•38,10•31)	0•423
**Onchocerca volvulus +ve**	14•7(61/414)	22•7(22/97)	1•30(0•70,2•41)	0•399
**HIV +ve**	8•3(50/606)	10•7(20/187)	1•71(0•93,3•15)	0•085

*OR adjusted for age, sex, education, marital status, employment and country

Supplementary Table 12: Risk factors for ACE in children after adjustment for a reduced set of confounders (age, sex and country)

	OR (95% CI) full model	OR (95% CI) reduced model	p value
**Seizures in the family**	1•72(1•31,2•25)	1•72(1•32,2•25)	<0•0001
**Maternal seizures**	2•84(1•24,6•49)	2•87(1•27,6•52)	0•011
**Abnormal delivery**	1•56(0•98,2•47)	1•48(0•95,2•32)	0•085
**Abnormal antenatal period**	2•15(1•53,3•02)	2•26(1•62,3•16)	<0•0001
**Home delivery**	1•24(0•97,1•57)	1•25(0•99,1•57)	0•060
**Problems after birth**	2•77(1•82,4•23)	2•98(1•97,4•52)	<0•0001
**Difficulties feeding, crying or breathing**	10•23(5•85,17•88)	10•53(6•04,18•34)	<0•0001
**Head injury**	1•97(1•28,3•03)	2•07(1•36,3•15)	0•0006
**Malnourished**	1•00(0•78,1•29)	1•00(0•78,1•27)	0•982
**Eats cassava**	0•99(0•77,1•26)	1•01(0•79,1•28)	0•951
**Dogs in household**	0•91(0•73,1•15)	0•89(0•72,1•11)	0•313
**Cats in household**	1•26(0•98,1•62)	1•29(1•02,1•65)	0•036
**Eats Pork**	0•99(0•79,1•25)	1•00(0•80,1•25)	0•990
**Malaria IgG +ve (schizont)**	1•14(0•75,1•73)	1•15(0•77,1•71)	0•504
**Hospitalised with malaria or fever**	2•01(1•17,3•45)	2•08(1•22,3•55)	0•007
**Toxocara canis IgG4 +ve**	1•19(0•85,1•66)	1•10(0•79,1•52)	0•565
**Toxoplasma gondii IgG +ve**	1•15(0•85,1•58)	1•20(0•89,1•63)	0•230
**Taenia solium +ve**	1•09(0•34,3•45)	0•93(0•31,2•80)	0•898
**Onchocerca volvulus +ve**	1•67(1•09,2•57)	1•58(1•04,2•40)	0•031
**HIV +ve**	1•28(0•80,2•03)	1•28(0•81,2•03)	0•281

Supplementary Table 13: Risk factors for ACE in adults after adjustment for a reduced set of confounders (age, sex and country)

	OR (95% CI) full model	OR (95% CI) reduced model	p value
**Seizures in the family**	2•30(1•73,3•07)	2•37(1•81,3•10)	<0•0001
**Maternal seizures**	3•02(0•75,12•14)	3•75(1•01,13•91)	0•049
**Abnormal delivery**	1•11(0•65,1•89)	1•02(0•63,1•67)	0•927
**Home delivery**	1•18(0•92,1•53)	1•23(0•97,1•55)	0•089
**Problems after birth**	6•41(3•28,12•53)	5•73(3•08,10•67)	<0•0001
**Head injury**	2•29(1•22,4•30)	1•90(1•05,3•45)	0•034
**Drinks alcohol**	0•88(0•67,1•15)	0•71(0•55,0•92)	0•009
**Eats cassava**	1•46(1•12,1•91)	1•38(1•08,1•76)	0•010
**Eats Pork**	1•10(0•89,1•36)	1•02(0•83,1•24)	0•882
**Uses drugs**	1•21(0•81,1•79)	0•90(0•62,1•31)	0•571
**Hypertension**	1•74(0•91,3•33)	1•82(0•98,3•36)	0•057
**Stroke**	1•94(0•61,6•15)	2•54(0•84,7•71)	0•099
**Diabetes mellitus**	2•48(0•56,10•87)	2•31(0•57,9•44)	0•242
**Malnourished**	1•25(0•93,1•67)	1•39(1•06,1•81)	0•018
**Dogs in household**	0•89(0•71,1•12)	0•82(0•67,1•02)	0•073
**Cats in household**	1•03(0•80,1•32)	1•01(0•80,1•28)	0•922
**Malaria IgG +ve (schizont)**	1•17(0•75,1•81)	1•16(0•77,1•75)	0•472
**Hospitalised with malaria or fever**	2•28(1•06,4•92)	2•23(1•07,4•65)	0•032
**Toxocara canis IgG4 +ve**	1•74(1•27,2•40)	1•86(1•39,2•48)	<0•0001
**Toxoplasma gondii IgG +ve**	1•39(1•05,1•84)	1•28(0•99,1•66)	0•057
**Taenia solium +ve**	1•98(0•72,5•43)	1•71(0•66,4•44)	0•270
**Onchocerca volvulus +ve**	2•23(1•56,3•19)	2•18(1•58,3•01)	<0•0001
**HIV +ve**	0•85(0•61,1•18)	0•90(0•66,1•22)	0•487
